# Supplementary material for: Decreased STARD10 Expression Is Associated with Defective Insulin Secretion in Humans and Mice
Source: Am J Hum Genet. 2017 Jan 26;100(2):238–56. doi: 10.1016/j.ajhg.2017.01.011 (PMC5294761; doi:10.1016/j.ajhg.2017.01.011)
Supplement: Document S1. Figures S1–S6 and Table S1 [file mmc1.pdf]

The American Journal of Human Genetics, Volume *100*

Supplemental Data

Decreased *STARD10* Expression Is Associated with Defective Insulin Secretion in Humans and Mice

Gaelle R. Carrat, Ming Hu, Marie-Sophie Nguyen-Tu, Pauline Chabosseau, Kyle J. Gaulton, Martijn van de Bunt, Afshan Siddiq, Mario Falchi, Matthias Thurner, Mickaël Canouil, Francois Pattou, Isabelle Leclerc, Timothy J. Pullen, Matthew C. Cane, Priyanka Prabhala, William Greenwald, Anke Schulte, Piero Marchetti, Mark Ibberson, Patrick E. MacDonald, Jocelyn E. Manning Fox, Anna L. Gloyn, Philippe Froguel, Michele Solimena, Mark I. McCarthy, and Guy A. Rutter

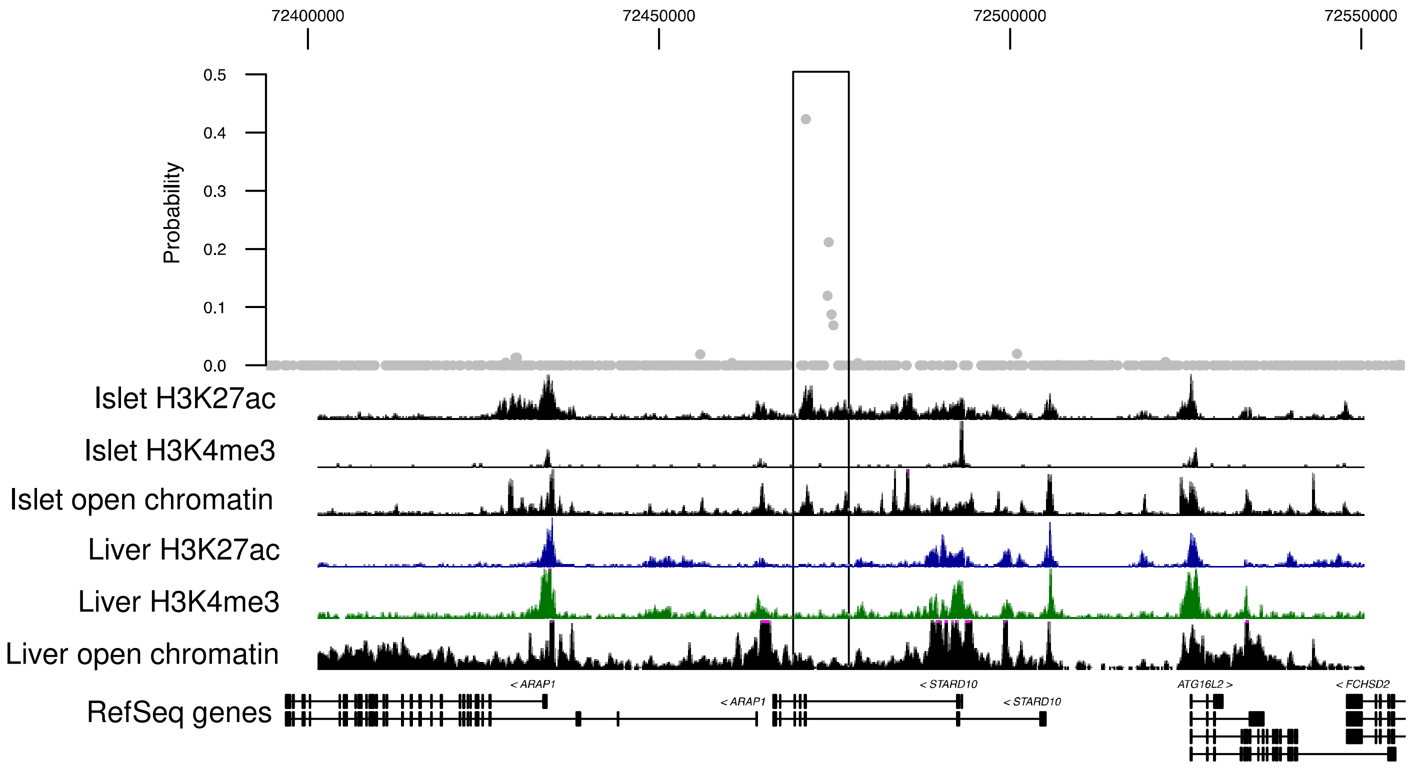
72400000
72450000
72500000
72550000
Probability
0.5
0.4
0.3
0.2
0.1
0.0
Islet H3K27ac
Islet H3K4me3
Islet open chromatin
Liver H3K27ac
Liver H3K4me3
Liver open chromatin
RefSeq genes
< ARAP1
< ARAP1
< STARD10
< STARD10
ATG16L2 >
< FCHSD2

Figure S1

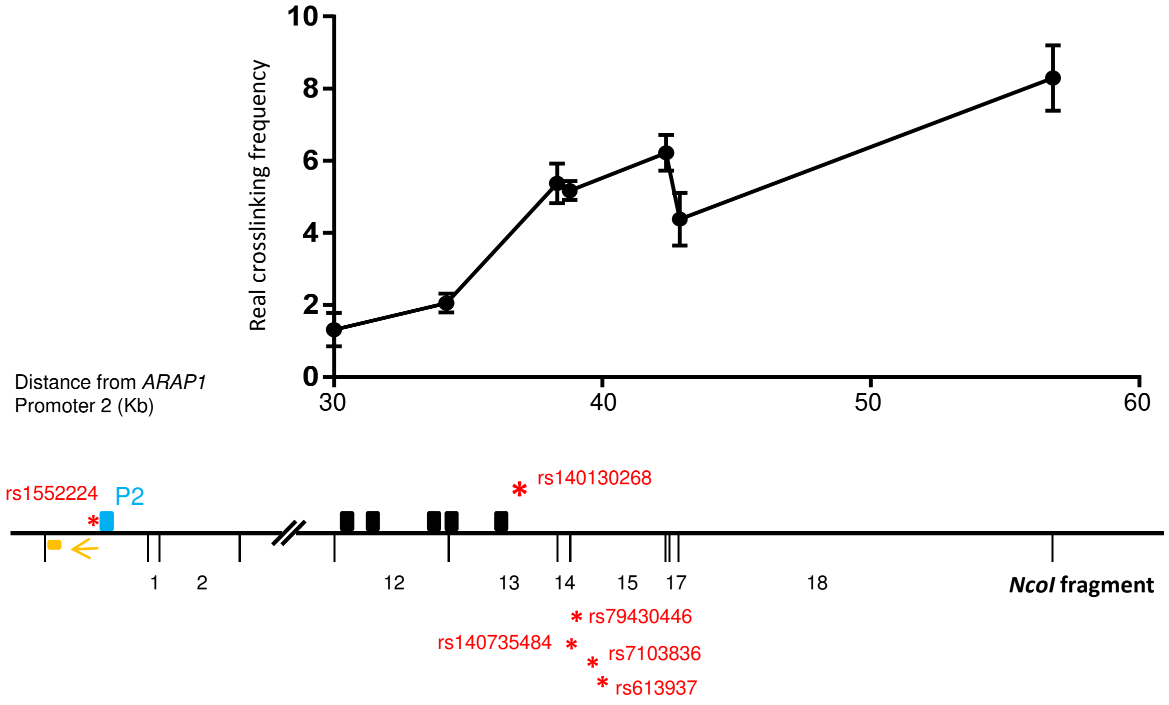
Real crosslinking frequency
10
8
6
4
2
0
Distance from *ARAP1* Promoter 2 (Kb)
30
40
50
60
rs1552224
P2
rs140130268
1
2
12
13
14
15
17
18
NcoI fragment
rs79430446
rs140735484
rs7103836
rs613937

Figure S2

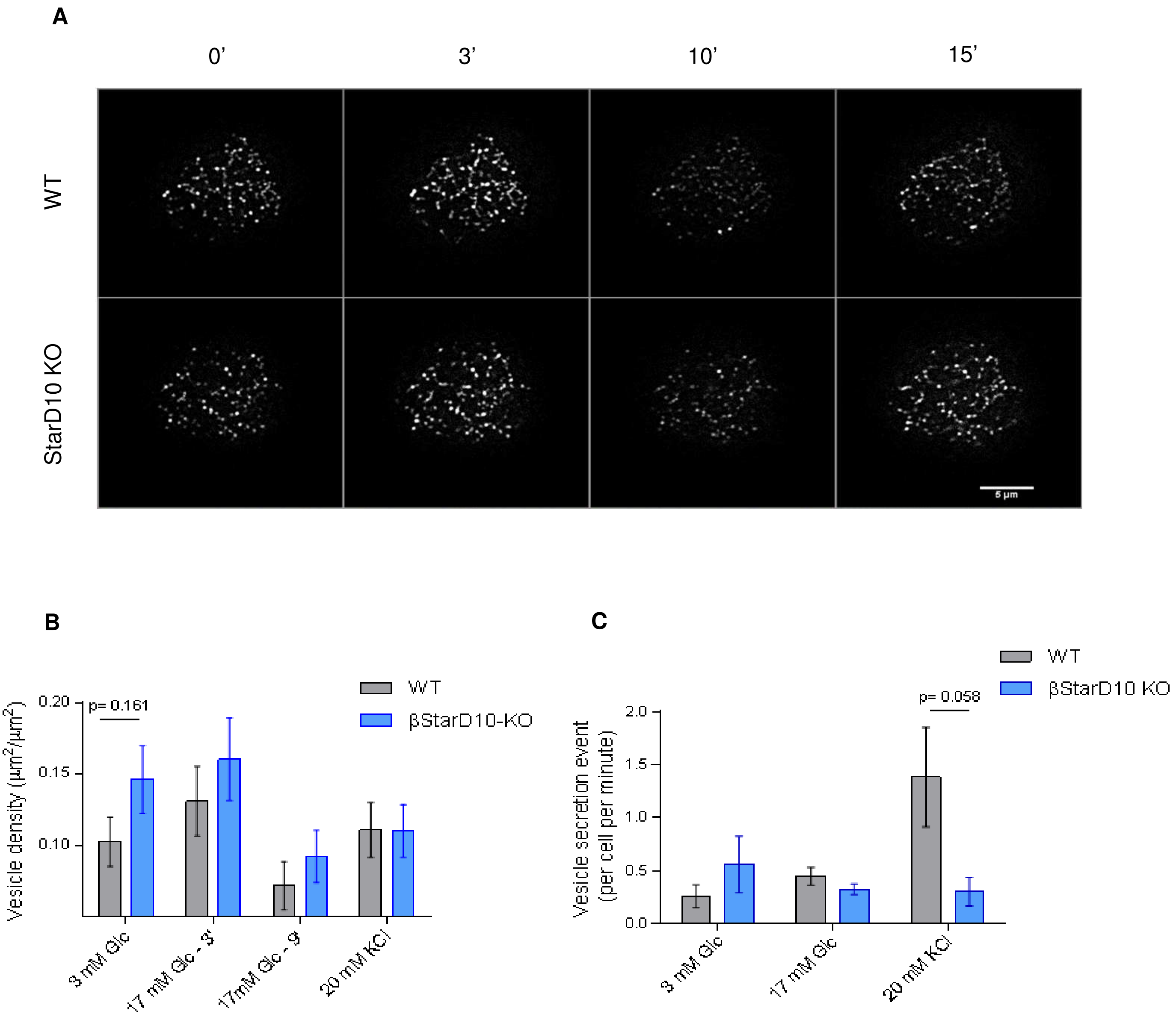
A
0'
3'
10'
15'
WT
StarD10 KO
5 µm
B
WT
βStarD10-KO
p= 0.161
Vesicle density (µm²/µm²)
0.20
0.15
0.10
3 mM Glc
17 mM Glc - 3'
17mM Glc - 9'
20 mM KCl
C
WT
βStarD10 KO
p= 0.058
Vesicle secretion event (per cell per minute)
2.0
1.5
1.0
0.5
0.0
3 mM Glc
17 mM Glc
20 mM KCl

Figure S3

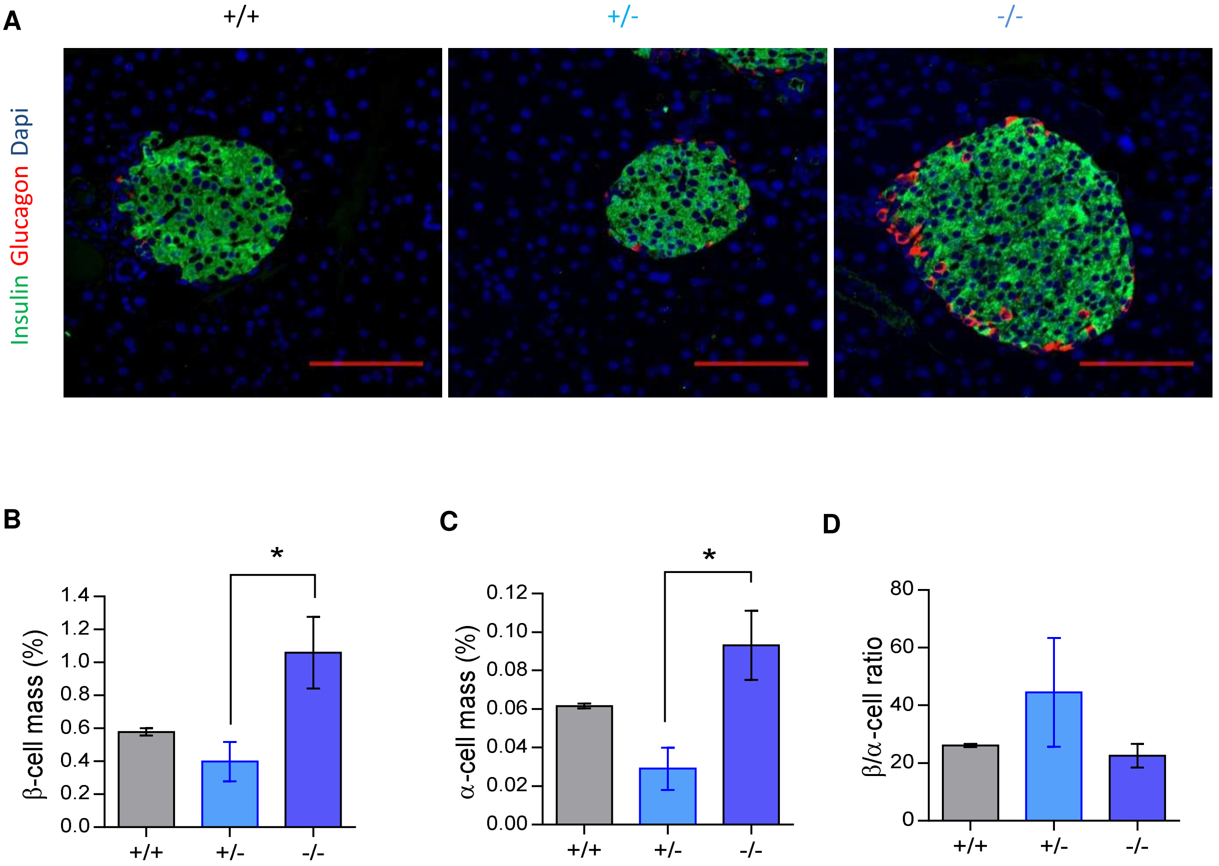
A
+/+
+/-
-/-
Insulin Glucagon Dapi
B
β-cell mass (%)
1.4
1.2
1.0
0.8
0.6
0.4
0.2
0.0
*
+/+
+/-
-/-
C
α-cell mass (%)
0.12
0.10
0.08
0.06
0.04
0.02
0.00
*
+/+
+/-
-/-
D
β/α-cell ratio
80
60
40
20
0
+/+
+/-
-/-

Figure S4

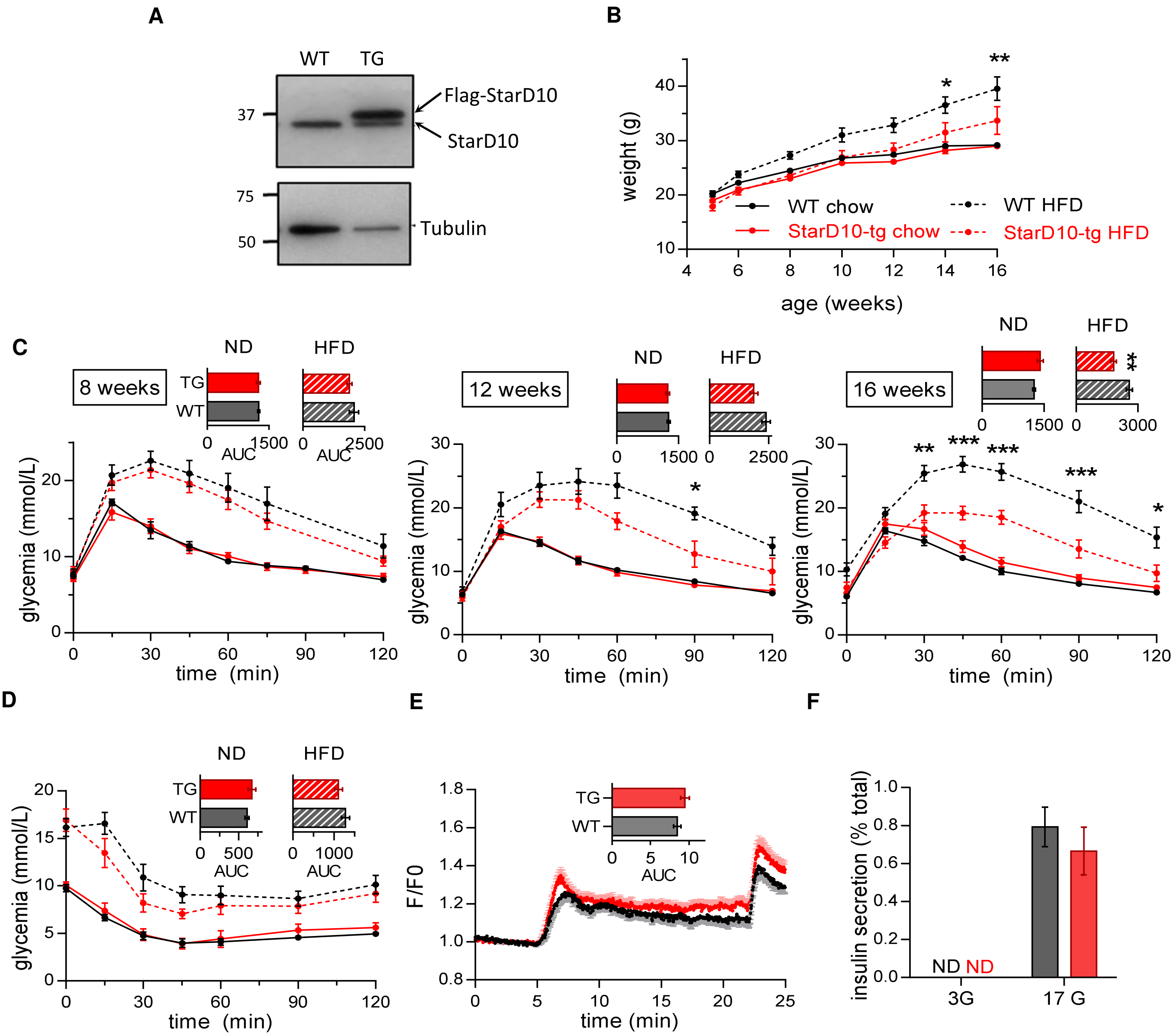
A
WT TG
37
Flag-StarD10
StarD10
75
50
Tubulin
B
weight (g)
age (weeks)
WT chow
StarD10-tg chow
WT HFD
StarD10-tg HFD
C
8 weeks
12 weeks
16 weeks
ND
HFD
TG
WT
AUC
glycemia (mmol/L)
time (min)
D
E
F
F/F0
time (min)
insulin secretion (% total)
ND ND
3G
17 G

Figure S5

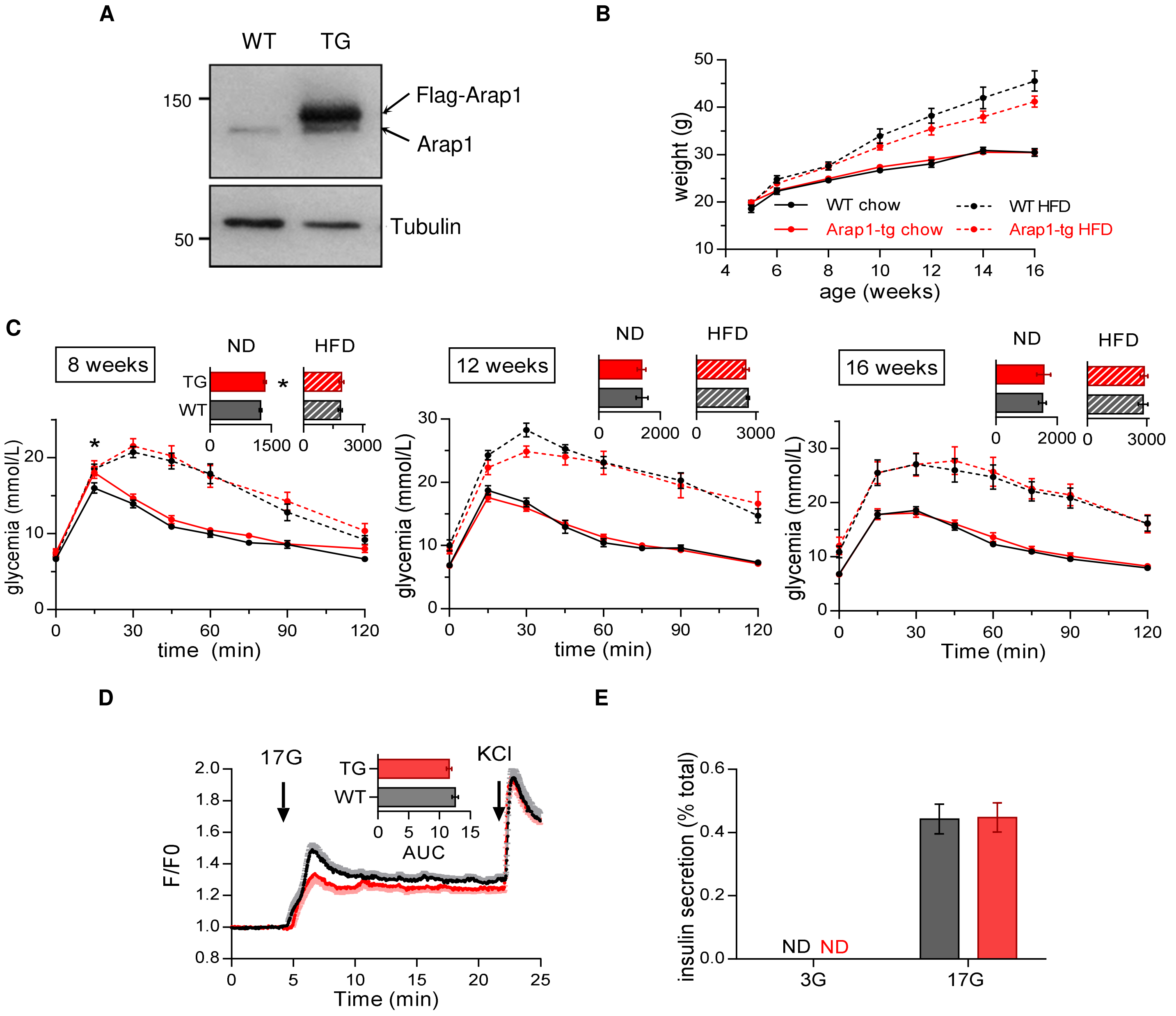

A
WT
TG
150
50
Flag-Arap1
Arap1
Tubulin
B
weight (g)
age (weeks)
WT chow
WT HFD
Arap1-tg chow
Arap1-tg HFD
C
8 weeks
12 weeks
16 weeks
ND
HFD
TG
WT
*
glycemia (mmol/L)
time (min)
Time (min)
D
17G
KCl
F/F0
AUC
Time (min)
E
insulin secretion (% total)
ND
ND
3G
17G

Figure S6

SUPPLEMENTAL DATA:

Supplemental figure legends:

Figure S1: **Epigenomic annotation at the *STARD10* locus**.

Top panel: posterior causal probabilities of each variant at the T2D signal. Bottom panel: raw signal from pancreatic islets and liver for H3K27ac, H3K4me3 and open chromatin (ATAC-seq or DNAase-seq). The variants most likely to be causal for the T2D signal (box) fall within a region of H3K27ac and open chromatin signal in islets, which are low for all assays in liver. Data are from ENCODE (The ENCODE Project Consortium, 2012)[1] and the NIH Roadmap 2015 (Roadmap Epigenomics Consortium, 2015) [2].

Figure S2: **A region carrying multiple risk variants is physically associated with the *ARAP1* promoter**.

3C-qPCR analysis of long-distance interactions at the *ARAP1-STARD10* locus assessed in human-derived EndoC-βH1 cells. The relative level of each ligation product (fragments 12 to 18) is plotted according to its distance (in Kb) from the *ARAP1* promoter 2 (P2). The constant primer and the Taqman probe are indicated in orange. Data were normalized to a *CXCL12* loading control. Blue box: *ARAP1* Promoter2, yellow box: qPCR probe; yellow arrow: qPCR constant primer; red stars: variants. The *NcoI* restriction fragments are indicated below the graph. *NcoI* fragments are numbered from fragment 1 to 18. The data represent the mean of three independent experiments.

Figure S3: **Secretory granule distribution and secretion assessed by TIRF Imaging**.

(A) Images were captured from single β-cells as described under "Supplemental methods" and show NPY-Venus-labelled granules before (0'), during (3') and after stimulation with 16.7 mM glucose. KCl (20 mM) was subsequently added at 12'. **(B)** Number of morphologically docked granules. Vesicle density was determined on top-hat filtered images as the surface occupied by vesicles on the total surface of the cell, and is related to the number of vesicles present at the membrane vicinity. Vesicle density was averaged on 120 images at 0, 3, 9 and 15 minutes for WT and β*StarD10* KO beta cells (*n*=6 and *n*=5 respectively). **(C)** Quantification of exocyotic events. Numbers of secretion events observable were counted per cell and per minutes for WT cells (n=5) and β*StarD10* KO cells (*n*=5) when imaged in 3mM Glc, 17mM Glc and 20mM KCl.

Figure S4: **Impact of *StarD10* deletion on β-cell mass**.

Pancreata from control (+/+), heterozygous (+/-) and homozygous (-/-) mice were fixed and subjected to immunohistochemical analysis for insulin and glucagon, as indicated. Representative islets are shown in (**A**; Scale bar: 100 µm). β-cell mass and α-cell mass represent the percentage of respectively insulin **(B)** and glucagon **(C)** positive staining area over the total pancreas section as measured using ImageJ software. β-cell/α-cell ratio was measured as the quantification of total insulin/glucagon positive staining on each pancreas section. **(D)** A total of five sections evenly separated by 25 µm in each pancreas was used (*p<0.05 vs +/-; *n*=3-4 animals in each group; One-way ANOVA, Tukey post-test).

Figure S5: **Improved glucose tolerance in *StarD10* transgenic mice.**

(A) Western blot analysis of *StarD10* expression showing the expression of the endogenous protein (lower band) and the flag-tagged transgene (upper band). **(B)** Growth curves of WT (black) and *StarD10* transgenic (red) male mice maintained on a regular chow diet (plain lines) or high fat diet (dotted lines) from 5 weeks of age. (*p<0.05, **p<0.01, WT vs StarD10-tg on high fat diet, two-way ANOVA, Sidak post-test). **(C)** Intraperitoneal glucose tolerance (1g/kg) was assessed at 8, 12 and 16 weeks of age in male mice. Areas under the curve are shown in inset. (*n*=5-11 animals per genotype. *p<0.05, **p<0.01, ***p<0.001, two-way ANOVA, Sidak post-test). **(D)** Intraperitoneal insulin tolerance was assessed at 17 weeks of age in mice fed a regular chow diet (0.75U/kg insulin), plain lines or a high fat diet (1U/kg), dotted lines. **(E)** Calcium responses to 17mM glucose and 20mM KCl of islets isolated from both male and female mice. **(F)** Insulin secretion assessed in islets isolated from both male and female, 19-week old WT (black) and littermate *StarD10* transgenic (red) mice on chow diet; ND: not detected; *n*=4-5 mice per genotype.

Figure S6: **No effect of *ARAP1* overexpression on glucose tolerance and insulin secretion in mice.**

(A) Western blot analysis of *Arap1* expression showing the expression of the endogenous protein (lower band) and the flag-tagged transgene (upper band). **(B)** Body weight of WT (black) and *ARAP1* transgenic (red) male mice maintained on a regular chow diet (plain lines) or high fat diet (dotted lines) from 5 weeks of age. **(C)** Intraperitoneal glucose tolerance (1g/kg) was assessed at 8, 12 and 16 weeks of age in male mice. Areas under the curve are shown in inset. *n*=9-12 animals per genotype. *p<0.05 (*: 15 min time point WT vs Arap1-tg, regular chow diet; two-way ANOVA, Sidak post-test). **(D)** Ca^{2+} responses of islets

isolated from both male and female mice to 17mM glucose and 20mM KCl. **(E)** Insulin secretion assessed in islets isolated from both male and female, 19-week old WT (black) and littermate *ARAP1* transgenic (red) mice on regular chow diet. ND: not detected. *n*=2 mice per genotype.

Table S1:

Primers for generating the *StarD10* expression construct

Primer name	Primer sequence
MmStarD10+Flag+NheI_F	tca**gctagc**atggactacaaggatgacgatgacaagGAAAAGCCAGCTGCCTCAACA
MmStarD10+XhoI_R	tca**ctcgag**TCAGGTGAGCGAGGTGTCAT

Primers for generating the *ARAP1* expression construct

HArap1+Flag+NheI_F	tca**gctagc**atggactacaaggatgacgatgacaagACCAAGAAGGAGGAGCCCCCA
HArap1+XhoI_R	tca**ctcgag**TCAGACGTTGCGCAGAAGAG

Genotyping primers:

StarD10 whole body KO mice:

STARD10-5arm-WTF	TGGGAAGCTCTTTGAGGGTA
Tm1a-5mut-R1	GAACTTCGGAATAGGAACTTCG
STARD10-Crit-WTR	TGGAAAATCAAATGCCCAAA

StarD10 conditional KO mice

STARD10-5arm-WTF	TGGGAAGCTCTTTGAGGGTA
STARD10-Crit-WTR	TGGAAAATCAAATGCCCAAA
Cre_F	CAGGCGTTTTCTGAGCATACC
Cre_R	CCGGTTATTCAACTTGCACCAT

StarD10 transgenic mice

StarD10_F	TGAGACTTTCGACATCGCCC
StarD10_R	CTTGGGACACCTCCAGGAAT
RIP7-rtTA_F	GGACGAGCTCCACTTAGACG
RIP7-rtTA_R	CAACATGTCCAGATCGAAATC

Arap1 conditional KO mice:

ARAP1-5arm-WTF	CCTCTTGGTCACTCCCATGT
ARAP1-Crit-WTR	ATCTCCACCCTCAACCTCCT
Cre_F	CAGGCGTTTTCTGAGCATACC
Cre_R	CCGGTTATTCAACTTGCACCAT

ARAP1 transgenic mice

ARAP1_F	TGCTTCTCAGACACGAACCA
ARAP1_R	CTTCCTCATCCACACTAAACACC
RIP7-rtTA_F	GGACGAGCTCCACTTAGACG
RIP7-rtTA_R	CAACATGTCCAGATCGAAATC

References

1. Gerstein MB, Kundaje A, Hariharan M, Landt SG, Yan KK, Cheng C, Mu XJ et al (2012) Architecture of the human regulatory network derived from ENCODE data. Nature 489 (7414):91-100

2. Kundaje A, Meuleman W, Ernst J, Bilenky M, Yen A, Heravi-Moussavi A, Kheradpour P et al (2015) Integrative analysis of 111 reference human epigenomes. Nature 518 (7539):317-330
