# Supplementary material for: Decreased STARD10 Expression Is Associated with Defective Insulin Secretion in Humans and Mice
Source: Am J Hum Genet. 2017 Jan 26;100(2):238–56. doi: 10.1016/j.ajhg.2017.01.011 (PMC5294761; doi:10.1016/j.ajhg.2017.01.011)
Supplement: Document S2. Article plus Supplemental Data [file mmc2.pdf]

ARTICLE

Decreased *STARD10* Expression Is Associated with Defective Insulin Secretion in Humans and Mice

Gaelle R. Carrat,[1] Ming Hu,[1] Marie-Sophie Nguyen-Tu,[1] Pauline Chabosseau,[1] Kyle J. Gaulton,[2,3] Martijn van de Bunt,[2,4] Afshan Siddiq,[5] Mario Falchi,[5,6] Matthias Thurner,[2,4] Mickaël Canouil,[7,8] Francois Pattou,[7,8] Isabelle Leclerc,[1] Timothy J. Pullen,[1] Matthew C. Cane,[1] Priyanka Prabhala,[1] William Greenwald,[2,3] Anke Schulte,[9] Piero Marchetti,[10] Mark Ibberson,[11] Patrick E. MacDonald,[12] Jocelyn E. Manning Fox,[12] Anna L. Gloyn,[2,4,13] Philippe Froguel,[5,7,8] Michele Solimena,[14,15,16] Mark I. McCarthy,[2,4,13] and Guy A. Rutter[1,*]

Genetic variants near *ARAP1* (*CENTD2*) and *STARD10* influence type 2 diabetes (T2D) risk. The risk alleles impair glucose-induced insulin secretion and, paradoxically but characteristically, are associated with decreased proinsulin:insulin ratios, indicating improved proinsulin conversion. Neither the identity of the causal variants nor the gene(s) through which risk is conferred have been firmly established. Whereas *ARAP1* encodes a GTPase activating protein, STARD10 is a member of the steroidogenic acute regulatory protein (StAR)-related lipid transfer protein family. By integrating genetic fine-mapping and epigenomic annotation data and performing promoter-reporter and chromatin conformational capture (3C) studies in β cell lines, we localize the causal variant(s) at this locus to a 5 kb region that overlaps a stretch-enhancer active in islets. This region contains several highly correlated T2D-risk variants, including the rs140130268 indel. Expression QTL analysis of islet transcriptomes from three independent subject groups demonstrated that T2D-risk allele carriers displayed reduced levels of *STARD10* mRNA, with no concomitant change in *ARAP1* mRNA levels. Correspondingly, β-cell-selective deletion of *StarD10* in mice led to impaired glucose-stimulated Ca^{2+} dynamics and insulin secretion and recapitulated the pattern of improved proinsulin processing observed at the human GWAS signal. Conversely, overexpression of *StarD10* in the adult β cell improved glucose tolerance in high fat-fed animals. In contrast, manipulation of *Arap1* in β cells had no impact on insulin secretion or proinsulin conversion in mice. This convergence of human and murine data provides compelling evidence that the T2D risk associated with variation at this locus is mediated through reduction in *STARD10* expression in the β cell.

Introduction

Normal glucose homeostasis depends on the correct processing of proinsulin and the storage of the mature hormone within secretory granules in the pancreatic β cell.[1] Stimulation of insulin secretion by glucose involves the uptake and metabolism of the sugar via glucose transporters (Glut2 and/or Glut1),[2] phosphorylation by glucokinase,[3] and enhanced mitochondrial ATP synthesis.[4,5] Closure of ATP-sensitive K^+ channels (K_{ATP}),[6] plasma membrane depolarization, and Ca^{2+} influx[7] then prompt the fusion of insulin-containing secretory granules with the plasma membrane. Additional, K_{ATP}-channel-independent mechanisms[8] also sensitize the secretory machinery to Ca^{2+}.[9]

Changes in both the number of β cells[10] and in the ability of these cells to respond to glucose[11] are involved in the development of type 2 diabetes (T2D [MIM: 125853]) in the face of insulin resistance.[12] In addition to lifestyle factors including obesity (MIM: 601665),[13] genetic factors also contribute substantially to overall T2D risk.[14,15] Genome-wide association studies have identified more than 90 loci associated with type 2 diabetes risk.[14,15] In the majority of cases the identified polymorphisms, which usually affect β cell function, lie in intronic or intergenic regions, and neither the identity of the responsible gene(s) nor the mechanism of action is clear.[15,16]

T2D is typically characterized by a disruption of proinsulin conversion[17,18] and carriers of T2D-risk alleles generally display increased proinsulin:insulin ratios compared with those who are homozygous for the protective allele.[19] By contrast, the T2D-risk alleles at a locus adjacent to *ARAP1* (MIM: 606646) (formerly called *CENTD2*) and *STARD10* on chromosome 11q13[20,21] are robustly associated with a

[1]Section of Cell Biology and Functional Genomics, Department of Medicine, Imperial College London, Hammersmith Hospital, du Cane Road, London W12 0NN, UK; [2]Wellcome Trust Centre for Human Genetics, University of Oxford, Roosevelt Drive, Oxford OX3 7BN, UK; [3]Department of Pediatrics, University of California San Diego, La Jolla, CA 92093, USA; [4]Oxford Centre for Diabetes, Endocrinology & Metabolism, University of Oxford, Oxford OX3 7LJ, UK; [5]Department of Genomics of Common Disease, Imperial College London, London W12 0NN, UK; [6]Department of Twin Research and Genetic Epidemiology, Kings College London, London SE1 7EH, UK; [7]European Genomic Institute for Diabetes (EGID), 59045 Lille, France; [8]Lille University, 59655 Villeneuve d'Ascq Cédex, France; [9]Sanofi-Aventis Deutschland GmbH, 65926 Frankfurt am Main, Germany; [10]Department of Endocrinology and Metabolism, University of Pisa, 56126 Pisa, Italy; [11]Vital-IT Group, SIB Swiss Institute of Bioinformatics, 1015 Lausanne, Switzerland; [12]Alberta Diabetes Institute Islet Core and Department of Pharmacology, University of Alberta, Edmonton, AB T6G 2E1, Canada; [13]Oxford NIHR Biomedical Research Centre, Churchill Hospital, Old Road, Headington, Oxford OX3 7LJ, UK; [14]Paul Langerhans Institute of the Helmholtz Center Munich at the University Hospital and Faculty of Medicine, TU Dresden, 01307 Dresden, Germany; [15]German Center for Diabetes Research (DZD e.V), 85764 Neuherberg, Germany; [16]Max Planck Institute of Molecular Cell Biology and Genetics, 01307 Dresden, Germany
*Correspondence: g.rutter@imperial.ac.uk
http://dx.doi.org/10.1016/j.ajhg.2017.01.011.
© 2017 The Author(s). This is an open access article under the CC BY license (http://creativecommons.org/licenses/by/4.0/).

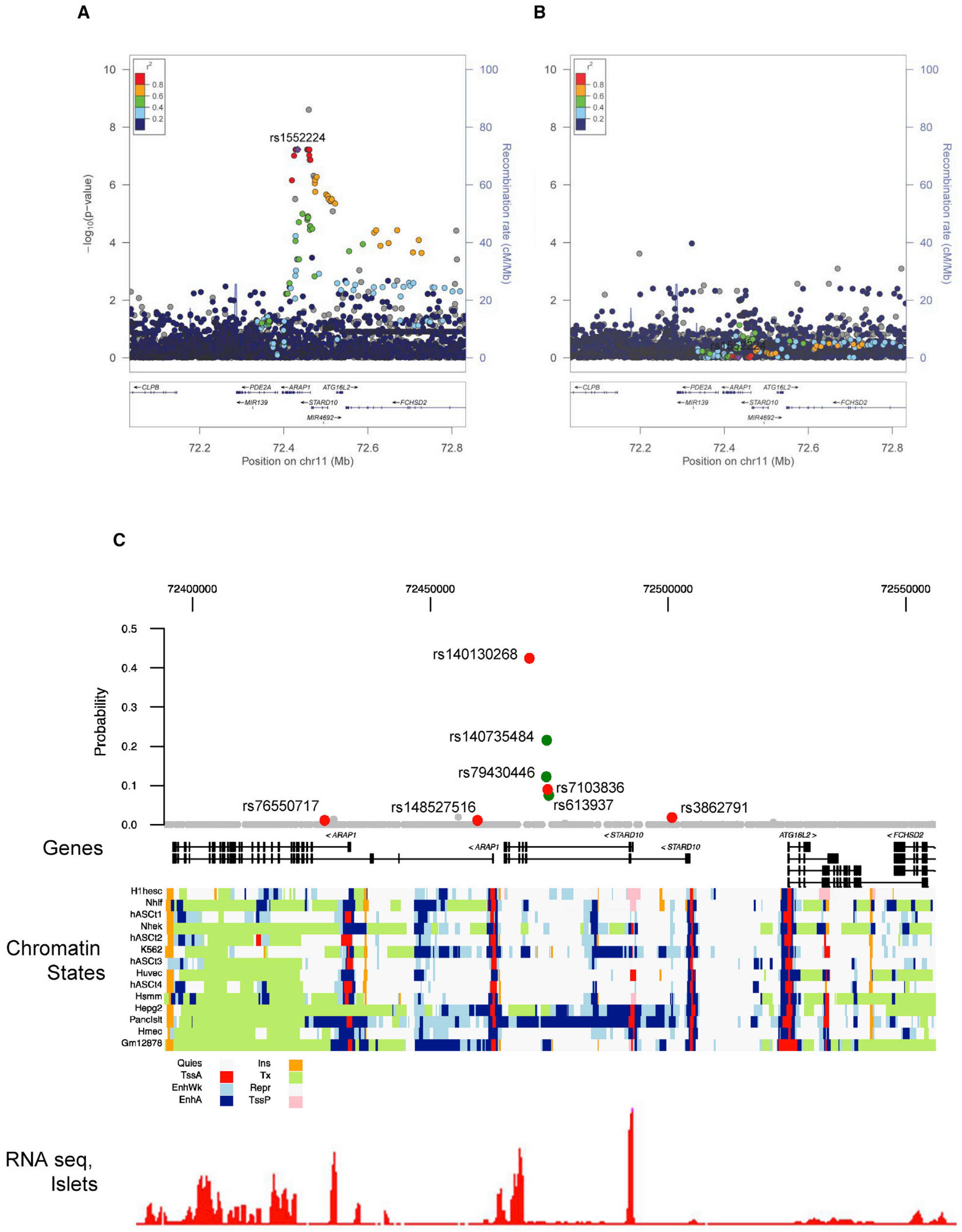

Figure 1. Identification of Likely Causal Variants at the *ARAP1/STARD10* Locus
(A and B) Quantitative trait locus association between variants at *ARAP1/STARD10* and *STARD10* (A) or *ARAP1* (B) expression level in human islets. Variants are strongly associated with *STARD10* expression level but not *ARAP1* level. Colors in each plot represent the extent of linkage disequilibrium between each tested variant and the T2D index variant

(legend continued on next page)

marked reduction in proinsulin:insulin ratios.[19,22] This unusual pattern implies preserved or improved proinsulin processing despite increased T2D risk.

ARAP1 encodes ARF-GAP, Rho-GAP, ankyrin repeat and pleckstrin homology domain-containing protein 1, or centaurin delta 2, an ArfGAP (GTPase activating protein) regulated by phosphatidyl inositol 1,4,5-*tris*phosphate. ARAP1 appears to act on ARF6 (MIM: 600464),[23] a known regulator of insulin secretion.[24] STARD10 (previously termed phosphatidylcholine transfer protein, PCTP-like) is a phospholipid transfer protein possessing a steroidogenic acute regulatory protein (StAR)-related lipid transfer ("StART") domain that facilitates the transport of phosphatidylcholine and phosphatidylethanolamine between intracellular membranes.[25] In the mouse, *StarD10* is strongly expressed in testes, liver, and kidney, but much more weakly in other tissues involved in insulin action and glucose metabolism such as skeletal muscle.[26] *STARD10* is the most strongly expressed of the genes close to the index SNP rs1552224 in human islets[19] and is also highly abundant (second centile of mRNAs) in mouse islets, where it is the most highly expressed StarD family member.[27] *STARD10* expression is also apparent in both human α and β cells, with similar mRNA levels in each cell type, and *ARAP1* is also expressed in both cell types albeit at levels approximately one-third those of *STARD10*.[28] Although global inactivation of *StarD10* in mice has previously implicated this protein in bile acid homeostasis,[29] its role in glucose homeostasis is unknown.

Recent expression quantitative trait loci (eQTL) studies in normoglycemic donors[30] have suggested that islet *STARD10* expression is correlated with T2D risk variants at this locus, whereas no such islet eQTL association was observed for *ARAP1*. These findings contrast with other results[31] reporting higher *ARAP1* mRNA synthesis from the T2D risk allele. Expression of other nearby genes at this locus—*FCHSD2* (MIM: 611565), *ATG16L2* (Figure 1C), and *PDE2A* (MIM: 602658) (not shown)—is relatively low in human islets.[19,28]

Here, we show first that disease-associated variants in this locus are associated with *STARD10*, but not *ARAP1*, mRNA levels in human pancreatic islets ascertained from both diabetic and non-diabetic individuals. Using genetic and genomic fine mapping and functional analysis in β cells, we identify a region in intron 2 of *STARD10* containing several variants that is likely to mediate T2D risk at this locus. Finally, we generate and characterize a series of mouse strains overexpressing, or inactivated selectively in the adult β cell, for *StarD10* or *Arap1*. These analyses reveal that *StarD10* is required for normal insulin secretion, though its deletion enhances proinsulin processing.

Material and Methods

Materials

cDNAs encoding human full-length *ARAP1* and mouse *StarD10* were purchased from Genscript and OriGene, respectively. Cell culture medium was from Sigma and fetal bovine serum (FBS) from SeraLab.

Identification of Causal Variants using MetaboChip and Functional Priors

We derived causal probabilities for each variant in the following way. We first obtained fine-mapping data of variants at 39 T2D loci (including *ARAP1*) from the Metabochip.[32] For each of the 49 distinct association signals at the 39 loci, we calculated the Bayesian posterior odds for all variants at each signal using the approach of Wakefield.[33]

Previous studies have demonstrated that sets of T2D risk loci share patterns of functional regulatory annotation in specific cell types and that this information can be used to help prioritize causal variants.[32,34–37] We thus obtained regulatory chromatin state data previously generated in 12 cell types which included 9 ENCODE cell types (Gm12878, HepG2, HUVEC, Hsmm, hESC, Hmec, NHLF, NHEK, and K562), pancreatic islets (PancIslt), and pre- and mature- adipocytes (hASCt1, hASCt4).[32,34,38,39] For each cell type, we used active enhancer (EnhA), weak enhancer (EnhWk), and active promoter (TssA) elements for a total of 36 chromatin annotations. For active enhancer elements, we further defined "stretch" enhancers using a previously described definition of active enhancers greater than 3 kb in size.[35]

We modeled the effect of these 36 annotations on the posterior odds of variants at the 39 T2D loci using fgwas.[40] In this procedure we first iteratively added annotations that increased the likelihood of the model. With this joint model, we selected the penalty with the highest penalized cross-validation likelihood. Using the optimal penalty, we maximized the cross-validation likelihood by iteratively removing annotations from the model. We then used the enrichment estimates of each annotation from this final model as functional priors to update the posterior odds for each variant at the *ARAP1/STARD10* locus. We finally calculated the posterior causal probability of each variant from these updated posterior odds.

Chromatin Accessibility Analysis

A total of 23 human islet samples were freshly isolated at the Oxford Centre for Islet Transplantation as described previously[30] and stored for 1–3 days in CMRL or in UW media. The latter were reactivated in CMRL for 1 hr before processing them further. Assay for transposase accessible chromatin (ATAC-seq) was performed on these 23 primary pancreatic islet samples as previously

(C) Top: probability that each variant at *ARAP1/STARD10* is causal for T2D risk using Metabochip fine-mapping and functional priors derived from chromatin state maps of 12 cell types from ENCODE, islets, and adipose tissue.[32] The indel rs140130268 has the highest causal probability ($\pi_c = 42\%$). Middle: chromatin states for each of the 12 cell types colored by state (abbreviations: Quies, quiescent; TssA, active promoter; EnhWk, weak enhancer; EnhA, active enhancer; Ins, insulator; Tx, transcription; Repr, repressed; TssP, poised promoter). Variants with highest causal probabilities, including rs140130268, fall in a stretch-enhancer region (dark blue) active in islets and largely inactive in other cell types. Variants highlighted in red were characterized individually in functional (promoter-luciferase) assays, those in green were analyzed in combination (see Results). Bottom: islet RNA-seq expression level of each gene transcript in the region.

described.[41] To remove primer dimers, the amplified libraries were additionally purified with Agencourt AMPure beads. Samples were multiplexed using primers Ad2.1-6 and paired-end sequenced using Illumina HiSeq 2500. Raw FASTQ reads were processed with a departmental/in-house pipeline[42] and on the DNase and ChIP pipeline website (Web Resources). Specifically, library/sequencing quality was checked with FASTQC (Web Resources) and reads were mapped to the human genome hg19 via bowtie.[43] For reads that could not be aligned the first time, adapters were removed at the 3′ end with Trim Galore (Web Resources). The resulting trimmed reads were then mapped again with bowtie. Any remaining unmapped and trimmed reads were processed with FLASH[44] and remapped a third time with bowtie. For each fine-mapped variant at the *ARAP1/STARD10* locus, we then re-mapped reads in the region against allele-specific reference genomes using WASP.[45] We then retained variants with at least five overlapping reads at the variant base from >2 different heterozygote samples. For the four resulting variants, we then tested for imbalance in the pooled read counts for each allele from heterozygote samples using a binomial test.

Expression Quantitative Trait Locus Analysis

IMIDIA Samples

Expression data were acquired and normalized from islet organ donors (81 healthy, 19 T2D) or partial pancreatectomy-laser microdissection samples (32 non-diabetic, 35 T2D) from the IMIDIA consortium (Web Resources; M. Solimena, personal communication) with appropriate permissions from donors and/or families. In brief, genotyping analysis was performed on the DNA from the same subjects using HumanOmni 2.5-8 beadchip from Illumina using a standard Infinium genotyping protocol. Standard quality control assessment was carried out on the genotyping data using PLINK (Web Resources).[46] *cis*-eQTL analysis was performed with a freely available program called matrix eQTL.[47] Linear model was used as a parameter for the analysis with age and gender as covariates. A "window size" of 100 kb was used as the cisDist; this distance represents the maximum distance at which gene-SNP pair is considered local. Only *cis*-eQTL results for *STARD10* and *ARAP1* are described in the present manuscript.

Oxford & Edmonton Samples

RNA-seq was performed on 174 human islet preparations collected in Oxford, UK, and Edmonton, Canada (an extension of the data reported in van de Bunt et al.[30]). In brief, samples were genotyped on Illumina HumanOmni2.5+Exome beadchips followed by imputation from the 1000 Genomes phase 3 panel using SHAPEIT2[48] and IMPUTE2.[49] Raw RNA-seq reads were aligned to the human genome reference hg19 with STAR[50] and expression quantified at the exon-level. Read count data was normalized to 20M reads, with exons with expression <1 count in >20% of all samples excluded. This was followed by rank normalization per exon, after which 30 hidden factors (accounting for non-genic variability in the samples) were inferred from the count matrix using PEER.[51] *cis*-eQTL analysis for *STARD10* and *ARAP1* was performed in a window flanking 1 Mb either side of the transcriptional start site using linear regression (with 30 PEER factors as covariates) implemented in FastQTL,[52] with p values adjusted through beta-approximation.

Liver Biopsies

Genotyping was performed using Illumina Metabochip DNA arrays.[53] mRNA levels were measured using the Illumina HumanHT-12 Expression Beadchip. *cis*-eQTL analysis was performed under R (v.3.3.1) using standard linear regression adjusted for age and BMI as implemented in FastQTL,[52] setting a maximum distance from the SNP location of 500 kB.

Animals and Ethics

All in vivo procedures were approved by the UK Home Office according to Animals (Scientific Procedures) Act 1986 (HO License PPL 70/7349) and were performed at the Central Biomedical Service, Imperial College, London, UK. Animals were housed 2 to 5 per individually ventilated cage in a pathogen-free facility with 12 hr light-dark cycle and had free access to standard mouse chow diet unless otherwise stated. For high-fat diet treatment, mice were placed on a high-fat diet at 5 weeks of age (DIO Rodent Purified Diet w/60% energy from fat; Test Diet). Human islet samples were obtained with appropriate local and ethical approval and consent from next of kin as required.

Generation of Transgenic Mice

Human *ARAP1* and murine *StarD10* coding sequences were amplified from *ARAP1-pcDNA3.1+* and *StarD10-pCMV-entry6*, respectively, with the addition of a single NH2 Flag tag by PCR, and inserted between the NheI and XhoI sites of the plasmid pBI-L Tet (Clontech). The resulting plasmid carried a bidirectional tetracycline-regulated promoter driving expression of both Flag:: *ARAP1* or Flag::*StarD10* and firefly luciferase.

The above expression cassette was excised from the plasmid backbone by *Aat*II and *Ase*I digestion and transferred by pronuclear microinjection into C57BL/6J mouse oocytes. Successful integrants were identified by PCR screening. *RIP7-rtTA* mice on a C57BL/6 background, expressing the reverse tetracycline transactivator under the control of the rat insulin promoter,[54] were crossed with transgenic mice to permit β-cell-specific, tetracycline-inducible expression of the transgene and luciferase.[55] Heterozygous transgenic mice were crossed to homozygous RIP7-rtTA mice to produce littermates of two genotypes: single transgenic (control, *RIP7-rtTA*/-) and double transgenic (*ARAP1* or *StarD10*-tg, *RIP7-rtTA*/*transgene-Luc*).

Generation of *StarD10*- and *Arap1*-Null Mice

StarD10 whole body and conditional knockout (KO) mice (C57BL/6NTac background) were generated by the trans-NIH Knock-Out Mouse Project (KOMP) and obtained from the KOMP Repository (Web Resources) via the international mouse phenotyping consortium (IMPC).[56] Mice homozygous for floxed *StarD10* (*StarD10*$^{fl/fl}$) or *Arap1* (*Arap1*$^{fl/fl}$) alleles were crossed to mice expressing *Cre* recombinase from the endogenous *Ins1* locus (*Ins1-Cre* mice).[57] This generated *StarD10*$^{fl/fl}$:*Ins1Cre*$^{+}$ (β*StarD10* KO) mice, where exon 3 was removed selectively by *Cre*-mediated excision in pancreatic β cells, or *Arap1*$^{fl/fl}$:*Ins1Cre*$^{+}$ (β*Arap1*KO) mice, where exon 12 of *Arap1* was removed. Genotyping was performed by PCR of DNA extracted from ear biopsies by the HotSHOT method[58] (primer sequences in Table S1). Ablation of gene expression from pancreatic islets was assessed by real-time quantitative PCR (qPCR) on islet RNA and western (immuno-) blotting.

In Vivo Physiological Studies

All studies were performed on male mice except when data were comparable between genders, in which case results from males and females were combined to gain statistical power, as indicated.

Intraperitoneal Glucose Tolerance Test

Mice fasted overnight (16 hr) were intraperitoneally injected at ~10 am with glucose, 1 g/kg mouse weight. Blood from the tail vein was obtained at 0, 15, 30, 45, 60, 90, and 120 min after injection. Blood glucose levels were measured with the Accu-Chek Aviva glucometer (Roche).

Plasma Insulin Measurement

Mice fasted overnight (16 hr) were intraperitoneally injected at ~10 am with 3 g glucose/kg mouse weight. Blood from the tail vein was collected into heparin-coated tubes (microvette) at 0, 15, and 30 min after injection. Plasma was separated by centrifugation at 2,000 × g for 5 min. Plasma insulin levels were measured using an ultrasensitive mouse insulin ELISA kit (Crystal Chem).

Insulin Tolerance Test

Human insulin (Actrapid, Novo Nordisk) was intraperitoneally injected at 3 pm into mice fasted for 5 hr. Blood glucose levels were measured at 0, 15, 30, 45, 60, 90, and 120 min after injection. The quantities of insulin injected were 0.5 U/kg (females, chow diet), 0.75 U/kg (males, chow diet and females, high fat diet), or 1 U/kg (males, high fat diet).

Proinsulin Measurement

Blood from the tail vein was collected into heparin-coated tubes from female and male mice fasted 5 hr. Plasma proinsulin levels were measured using a rat/mouse proinsulin ELISA kit (Mercodia).

Islet Isolation and Culture

Mouse islets were isolated after collagenase digestion (Collagenase NB8 Broad Range, 1 mg/mL, Serva Electrophoresis)[59] and subsequently cultured in RPMI1640 medium, containing 11 mM glucose (Sigma) and supplemented with 10% (v/v) fetal bovine serum plus penicillin (100 units/mL) and streptomycin (0.1 mg/mL) at 37°C in an atmosphere of humidified air (95%) and CO_2 (5%), for 2 or 3 days prior to experiments.

Insulin Secretion from Mouse Islets

Islets (10/well) were incubated in triplicate for each condition and treatment. Islets were pre-incubated for 1 hr in 3 mM glucose Krebs-Ringer-HEPES-Bicarbonate (KRH) buffer prior to secretion assay (30 min) in 3 mM, 17 mM glucose, or 30 mM KCl. The supernatant were collected and the islets were lysed in 1 mL of acidified ethanol to measure total insulin content. The insulin concentration was measured by radioimmunoassay (LINCO/Millipore) or using an HTRF kit (Cisbio Bioassays).[59]

qRT-PCR

Total cellular RNA from mouse islets or other tissues was obtained using TRIzol reagent (Invitrogen) and reverse transcribed with a High-Capacity cDNA Reverse Transcription Kit (Applied Biosystems) according to the manufacturer's instructions. Real-time PCR was performed on an ABI-Prism Fast 7500 device (Applied Biosystems) using the Fast SYBR Green Master Mix (Applied Biosystems).

Promoter-Reporter Assays and Chromatin Conformation Capture

INS1(832/13) pancreatic β cells[60] were cultured in RPMI medium (11 mM glucose) supplemented with 10% (v/v) fetal calf serum, 20 mM HEPES, 50 μM beta-mercaptoethanol plus penicillin (100 units/mL) and streptomycin (0.1 mg/mL) at 37°C in an atmosphere of humidified air (95%) and CO_2 (5%). EndoC-βH1 cells were kindly provided by Dr. Philippe Ravassard (CRICM CNRS UMR 7225, Paris, France) and grown in serum-free DMEM (Life Technology) containing low glucose (1 g/L), 2% (w/v) albumin from bovine serum fraction V (Roche Diagnostics), 50 μM β-mercaptoethanol, 10 mM nicotinamide (VWR), 5.5 μg/mL transferrin (Sigma-Aldrich), 6.7 ng/mL sodium selenite (Sigma-Aldrich), penicillin (100 units/mL), and streptomycin (100 μg/mL).

Identification and Cloning of the ARAP1/STARD10 Variant-Bearing Regions

Genomic DNAs from HEK293 cells were extracted using DNeasy blood and tissue kit according to manufacturer's instruction (QIAGEN). PCR reactions using Phusion high-fidelity DNA polymerase (New England Biolabs) were carried out to amplify genomic DNA fragments carrying variants using the primer sets as follow: rs148527516: forward: 5′-GCTGCGTCGACGG CCTGGTCCACCACTAGCC-3′ and reverse: 5′-GCAAGGGATC CGCCTCCTACTCAACCCCAGC-3′; rs140130268: forward: 5′-GC TGCGTCGACCAGCTCCCCAAAAAGCCACC-3′ and reverse: 5′-GCAAGGGATCCCGGGTGTGGTGGCTGACACC-3′; rs3862791: forward: 5′-ACTGAGGATCCGCTGCGTCGACCCGCGTGAGGA CTGGTGTGG-3′ and reverse: 5′-ACTGAGTCGACGCAAGGGAT CCGCCTCCTGACTTCAGGTGAGG-3′; rs7103836: forward: 5′-AC TGAGGATCCAGAGAAGCCTGGCAAATAGCACC-3′ and reverse: 5′-ACTGAGTCGACGCTGTTTGGATGCTAACGATGATGC-3′; rs76550717: forward: 5′-ACTGAGGATCCAATCTGGGGCCAAGG GGTGG-3′ and reverse: 5′-ACTGAGTCGACGAGCCAGGCTC CCTCAATCC-3′. PCR products were gel purified, digested with BamHI and SalI, and sub-cloned into the pGL3-promoter vector (Promega). To generate allelic variants, site-specific mutagenesis was carried out by a PCR-based method (Q5 site-directed mutagenesis kit, New England Biolabs) according to the manufacturer's instructions. All constructs were subjected to DNA sequencing.

Luciferase Assay

Luciferase constructs containing variant DNA fragments of 500–600 bp in each case were co-transfected, using Lipofectamine 2000 (Life Technologies), with CMV-Renilla construct as internal control, into INS1 (832/13) cells according to manufacturer's instruction. After 48 hr, transfected cells were washed once with PBS and lysed directly in passive cell lysis buffer from Luciferase Assay System (Promega). Cells were incubated on a rotating platform at room temperature for 10 min to ensure complete lysis of cells, and then spun at 10,000 rpm for 1 min to remove cell debris. Luciferase activity was determined with the Dual-luciferase Assay Reporter System on a Lumat LB9507 luminometer (Berthold Technologies).

Chromosome Conformation Capture

Assays were carried out as described.[61] In brief, a suspension of EndoC-βH1 cells was cross-linked with 2% (v/v) formaldehyde at room temperature for 10 min. The cross-linked DNA was digested overnight with NcoI. DNA fragments were ligated with T4 DNA ligase at 16°C overnight (14–16 hr). The ligated 3C DNA was purified by extraction with phenol/chloroform and precipitation with ethanol. The ligation products were quantitated by real-time PCR and normalized to the human *CXCL12*. The standard curve for each primer pair was generated using NcoI-digested and T4 DNA-ligated BAC DNA (RP11-101P7) encompassing the human *ARAP1*, *STARD10*, and *ATG16L2* loci. The constant primer was located in the promoter 2 region of *STARD10* and its DNA sequence was 5′-CGGAGCCTCCGCGGAGGACC-3′. The sequence of the qPCR probe was 5′-CGCTTCACCTGGCTGGG GAGTGGCTCCTAG-3′. The probe was labeled with both FAM and TAMRA. The individual primers covering variant regions were: for NcoI fragment −4: 5′-GCAGCTTATCTCAGAT

Table 1. Association of *cis*-eQTLs with rs1552224 and rs11603334

SNP	Gene	Probe	OD Control		OD All		LCM Control		LCM All	
			β (SE)	p Value	β (SE)	p Value	β (SE)	p Value	β (SE)	p Value
rs1552224	*STARD10*	223103_at	0.26 (0.10)	0.012*	0.30 (0.09)	0.0023*	0.22 (0.13)	0.10	0.32 (0.10)	0.0025*
		232322_x_at	0.16 (0.09)	0.069	0.19 (0.08)	0.025*	0.20 (0.13)	0.13	0.21 (0.10)	0.039*
	ARAP1	34206_at	0.12 (0.08)	0.11	0.07 (0.08)	0.36	0.06 (0.09)	0.51	–0.04 (0.07)	0.65
		212516_at	0.11 (0.06)	0.10	0.06 (0.06)	0.33	0.04 (0.08)	0.59	–0.07 (0.07)	0.26
rs11603334	*STARD10*	223103_at	0.26 (0.10)	0.013*	0.30 (0.09)	0.0023*	0.22 (0.13)	0.10	0.32 (0.10)	0.0025*
		232322_x_at	0.16 (0.09)	0.067	0.19 (0.08)	0.023*	0.20 (0.13)	0.13	0.21 (0.10)	0.039*
	ARAP1	34206_at	0.13 (0.08)	0.095	0.07 (0.08)	0.33	0.06 (0.09)	0.51	–0.04 (0.07)	0.65
		212516_at	0.11 (0.06)	0.093	0.07 (0.06)	0.29	0.04 (0.08)	0.58	–0.08 (0.07)	0.27
		225883_at	–	–	–	–	0.05 (0.11)	0.62	–0.05 (0.08)	0.57

Probe names are from Human Genome U133 Plus 2.0 Array from Affymetrix. p values calculated by linear model, and beta (β) is measuring the effect size estimate. All subjects were corrected for age and gender as covariates in the analysis. Significant values indicated by asterisk (*).

TGAGCCC-3′; fragment −6: 5′-CCGTGATGTCATCACCCTCC-3′; fragment −7: 5′-CCCAACCTTTTTGGCACCAGG-3′ and fragment −8: 5′-GCACAGCTTAGGAAGGGTCTC-3′C. The Taqman reaction was carried out on fast 9700 PCR machine with Taqman Fast advanced reagents. PCR reactions were set as below: 95°C for 10 min, then with 45 cycles at 95°C 30 s and 58°C 45 s. The real crosslinking frequencies were plotted as percentage of that of the human *CXCL12*.

To assess the chromatin association between *ARAP1* promoter region and the variants, the constant primer was designed to locate in the promoter 2 region of *ARAP1* and its DNA sequence was 5′-ACTTCTGTGAGCTCCCTGAGG-3′. The sequence of the qPCR probe was 5′-CCAGGCCTGGCCCTGTGCTGGCTCCT GAGG-3′. The probe was labeled with both FAM and TAMRA. The individual primers covering the variants regions were: for NcoI fragment 12, 5′-CCCAACCTTTTTGGCACCAGG-3′; fragment 13, 5′-CCGTGATGTCATCACCCTCC-3′; fragment 14, 5′-CCTCCTGCACTGAGATTCTCC-3′; fragment 15, 5′-GCAGCT TATCTCAGATTGAGCCC-3′; fragment 17, 5′-CCTGGGTCCC TAGGACTTTGG-3′; and fragment 18, 5′-CTGGCAGAGGTGGTTT GAGC-3′

Statistical Analysis

Data are expressed as means ± SEM. Significance was tested by Student's two-tailed t test, Mann-Whitney test for non-parametric data, and one- or two-way ANOVA with SIDAK multiple comparison test, as appropriate, using Graphpad Prism software. $p < 0.05$ was considered significant.

Results

cis-eQTL Analysis Reveals Association between T2D Risk Variants at the *ARAP1/STARD10* Locus and *STARD10* but Not *ARAP1* Expression

We first explored the possibility that possession of risk alleles at this locus may lead to changes in the expression of *STARD10* or *ARAP1* in human islets from three separate sources. First, we examined two cohorts from a recently described biorepository from the IMIDIA consortium (M. Solimena, personal communication). This consists of samples from non-diabetic and T2D subjects taken either (1) post-mortem (organ donors; OD) or (2) after partial pancreatectomy for pancreatic disease and laser capture microdissection (PP-LCM) (167 samples in total). Associations were determined between signals from the microarray expression probes indicated in Table 1 and the previously defined SNPs at this locus, rs1552224 and rs11603334,[19–21] which were used as proxies for the likely causal variants (see below) with which they are in linkage disequilibrium (LD). We note that although *cis*-eQTLs detected with this approach are likely to reflect associations with β cells, we do not exclude a contribution from other islet endocrine cells to the observed signals.

Significant associations were detected between *STARD10* mRNA levels and genotype in both OD and PP-LCM groups, irrespective of the SNP analyzed (Table 1). In all analyses, increased expression was associated with possession of the minor (T2D-protective) allele.[19–21] By contrast, no such associations were apparent for *ARAP1* expression (Table 1). In RNA-seq data from human islet preparations ascertained from 174 normoglycemic ODs in Oxford and Edmonton (this is an extension of a recently reported sample)[30] the lead variants at the locus were full LD proxies for the significant (FDR < 1%) *cis*-eQTL for *STARD10* (Figure 1A), but no association with *ARAP1* (Figure 1B) mRNA levels was observed.

In the OD and PP-LCM islets from the IMIDIA samples, it was possible to compare *STARD10* and *ARAP1* expression levels from islets gathered from T2D (n = 54) and nondiabetic (n = 113) subjects. In both OD and PP-LCM, T2D individuals displayed reduced *STARD10* expression; whereas a reduction in *ARAP1* expression in T2D was observed in OD but not PP-LCM subjects (Table 2).

Fine Mapping of Variants at the *ARAP1/STARD10* Locus

We next used data from a dense fine-mapping study of 39 T2D loci on the Metabochip, involving 27.2k cases and

Table 2. Expression of *STARD10* and *ARAP1* in Healthy and T2D Islets

	OD Data				LCM Data			
Gene	Probeset	Log Fold Change	p Value	Adjusted p Value	Probeset	Log Fold Change	p Value	Adjusted p Value
STARD10	STARD10_238911_at	–0.27	0.00618	0.0185*	STARD10_238911_at	–0.37	0.0216	0.0862
	STARD10_232322_x_at	–0.15	0.00496	0.0149*	STARD10_223103_at	–0.17	0.00211	0.00846*
ARAP1	ARAP1_212516_at	–0.25	0.000479	0.00144*	ARAP1_34206_at	0.10	0.0658	0.263
					ARAP1_225883_at	0.17	0.265	1.06

Data are from organ (OD, n = 81 and 19 normoglycemic and T2D, respectively) and partial pancreatectomy/laser capture microdissection (LCM, n = 32,35) donors (M. Solimena, personnal communication). Fold changes indicate the rate of expression in T2D versus non-diabetic islets. Significant values indicated by asterisk (*).

57.6k controls,[32] to determine which variants at the *ARAP1/STARD10* locus were most likely to be causal. First, using genetic data alone, we calculated the Bayesian posterior causal probability (π_c) for each variant and identified the set of those variants that collectively explained 99% of the total probability. This "credible set" included 27 variants, each with relatively modest probabilities of being causal (max $\pi_c = 0.13$).

To further distinguish between these 27 candidate variants, we used the fgwas approach[40] to integrate the T2D fine-mapping data with chromatin state maps from 12 human cell types (including islets; see Material and Methods). We determined the degree of enrichment for each enhancer and promoter annotation with respect to T2D association data across all 39 T2D loci for which high-density genotype data were available on Metabochip (see Material and Methods) and used these enrichment estimates as a prior on the causal evidence for each variant at the *ARAP1/STARD10* locus specifically. The joint analysis reduced the 99% credible set to 12 variants and identified several variants with high posterior probabilities (Figure 1C). These high probability variants all map to a 5 kb interval in intron 2 of *STARD10* within a 22.6 kb region of stretch-enhancer elements active in pancreatic islets. Of the variants within this set, the rs140130268 indel accounted for almost half of the re-weighted causal probability ($\pi_c = 42.3\%$). Neither the previously reported "index" SNP (rs1552224)[20,62] nor a second SNP (rs11603334) previously assigned a putative functional role[31] were members of this re-weighted credible set (both have $\pi_c < 1 \times 10^{-5}$), indicating that these specific variants are likely to have been proxies for the true causal variant, rather than being directly responsible themselves. The region surrounding the 5 kb region of interest was relatively more inert in other cell types including in HepG2 hepatoma cells (Figure 1C). Correspondingly, more detailed scrutiny of activating H3K4me3 and H3K27ac signals, as well as open chromatin, in both islet and liver, confirmed the differences between these two tissues in the region hosting the five variants (Figure S1), in line with an absence of *cis*-eQTLs for *ARAP1* or *STARD10* in liver, as described below.

To next determine whether variants in the 5 kb fine-mapped region influence local chromatin structure, we obtained chromatin accessibility data in primary pancreatic islets using ATAC-seq.[41] We identified four fine-mapped variants in the 5 kb interval that directly overlapped regions of accessible chromatin and thus could serve as markers of allelic activity. For each variant, we tested for allelic imbalance in ATAC-seq signal in samples heterozygous for all of these variants. We identified evidence for imbalance at three variants (rs7103836 $p = 3.0 \times 10^{-6}$, rs3862791 $p = 3.5 \times 10^{-3}$, rs76550717, $p = 0.02$). At all three variants, the T2D risk-increasing allele was correlated with lower chromatin accessibility, consistent with the correlation of T2D risk alleles with lower *STARD10* expression. This demonstrates that risk alleles of variants in the 5 kb fine-mapped region are correlated with decreased islet chromatin accessibility in islets and implies that one or several variants in this region directly affects local chromatin structure. Note that indel imbalance could not be assessed accurately given asymmetric read and mapping efficiencies.

Indel rs140130268 (Figure 1C), which had the strongest posterior causal probability, as well as other variants in the credible set, were carried forward for functional analysis: two other variants, rs3862791 and rs148527516 (Figure 1C), with low probabilities of contributing to disease risk (rs3862791 $\pi_c = 0.02$, rs148527516 $\pi_c = 0.0001$), served as negative controls. Promoter-reporter studies were performed in the insulin-secreting rat INS1(832/13) cell line.[60] Regions of ~0.5 kb around the human variants were PCR amplified and sub-cloned into plasmids downstream of firefly luciferase cDNA expressed under the control of an SV40 promoter (Figure 2B). Co-transfection with a control vector allowed expression to be normalized to that of *Renilla* luciferase. No differences were observed between the apparent enhancer activity of risk and protective alleles of rs148527516, rs3862791, rs7103836, or rs76550717. Moreover, simultaneous replacement of the two risk for the two protective alleles at the closely neighboring (~650 bp apart) variants rs79430446 and rs140735484, or of those at rs7103836 and rs61397, failed to impact

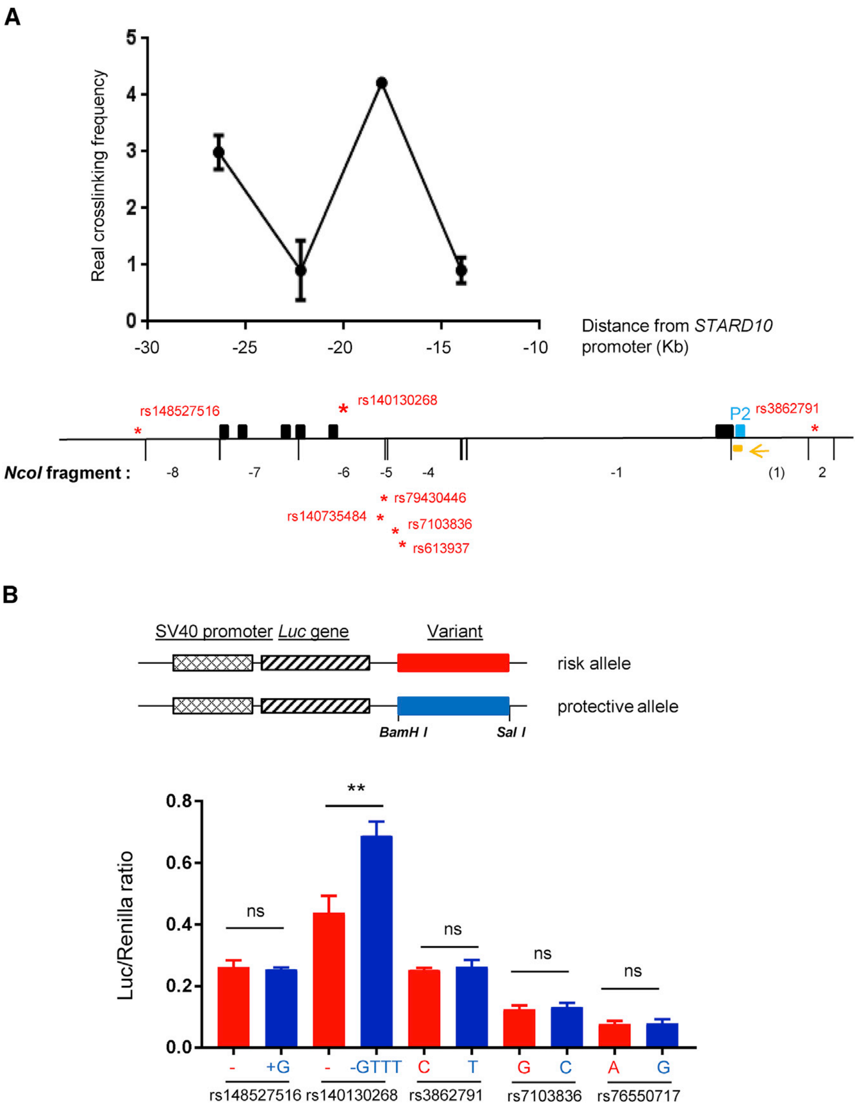

Figure 2. A Region Carrying Multiple Risk Variants Including rs140130268 Is Physically Associated with *STARD10* Promoter and Influences Enhancer Activity

(A) 3C-qPCR analysis of long-distance interactions at the *STARD10* locus assessed in human-derived EndoC-βH1 cells. The relative level of each ligation product (fragments −4 to –7) is plotted according to its distance (in kb) from the *STARD10* promoter 2 (P2). The constant primer and the Taqman probe are indicated in orange. Data were normalized to a *CXCL12* loading control. Blue box, *STARD10* Promoter2; yellow box, qPCR probe; yellow arrow, qPCR constant primer; red stars, variants. The NcoI restriction fragments are indicated below the graph. NcoI fragments are numbered from fragment –1 to –8. The data represent two or three independent experiments.

(B) The protective allele of variant rs140130268 increases the transcriptional activity of the corresponding region in insulin-secreting cells by luciferase-reporter assay. Diagram of the luciferase reporter constructs carrying either the risk or protective variants. Transcriptional activities measured by dual luciferase assay after transfection of INS1(832/13) cells (see Material and Methods). The risk variant is shown in red and the protective variant in blue. Data are from five independent experiments, represented as the mean ± SEM, and significance was calculated by Mann-Whitney two-tailed test.

enhancer activity in this assay (not shown). By contast, the T2D-protective (-GTTT) allele at rs140130268 displayed significantly (~40%) higher activity than the T2D-risk form (Figure 2B).

To determine whether the above region was able to physically associate with the *STARD10* promoter, chromatin conformation capture (3C) analysis[61] was performed using human EndoC-βH1 cells.[63] *STARD10* may be expressed from one of two promoters (P1 and P2) located at the 5′ end of exon 1 or exon 2, respectively.[35] RNA-seq analysis from human islets (Figure 1C)[35] indicates that transcripts from P2 are the most abundant in islets. The results of 3C analysis using the restriction enzyme NcoI are presented in Figure 2A. Cross-linking frequencies were observed at the genomic DNA fragment (NcoI fragment –4) carrying rs140130268 as well as surrounding regions (NcoI fragments –4 and –7) demonstrating that *STARD10* P2 interacts physically with the 5 kb fine-mapped interval and may thus be impacted by variation at rs140130268 as well as other credible set variants.

A similar experiment was performed with *ARAP1* promoter 2, which is the most active in islets (Figure S2). We also observed an association between the previous index SNP at this locus (rs1552224) which is located in the 5′ UTR of *ARAP1* and sites located across the entire locus.

cis-eQTLs for *STARD10* and *ARAP1* Are Not Detected in the Liver

Given the characteristic effect of this locus on apparent proinsulin processing and the importance of liver, where *STARD10* is also highly expressed, for proinsulin clearance, we assessed data from previous reports[64,65] to determine whether similar T2D-GWAS coincident *cis*-eQTLs were observed for this tissue. We examined publicly available datasets including samples from 97 (GTex)[64] and 600 (STARNET)[65] samples, respectively. In contrast to the situation in islets, the previously reported *cis*-eQTLs for *STARD10* in the liver were in very low LD (r^2 ~0.1) with the identified T2D locus, and no liver *cis*-eQTL was observed for *ARAP1*.

In order to further validate the above results, we searched for *cis*-eQTLs for these genes in a separate cohort of liver biopsies from 186 female subjects in the

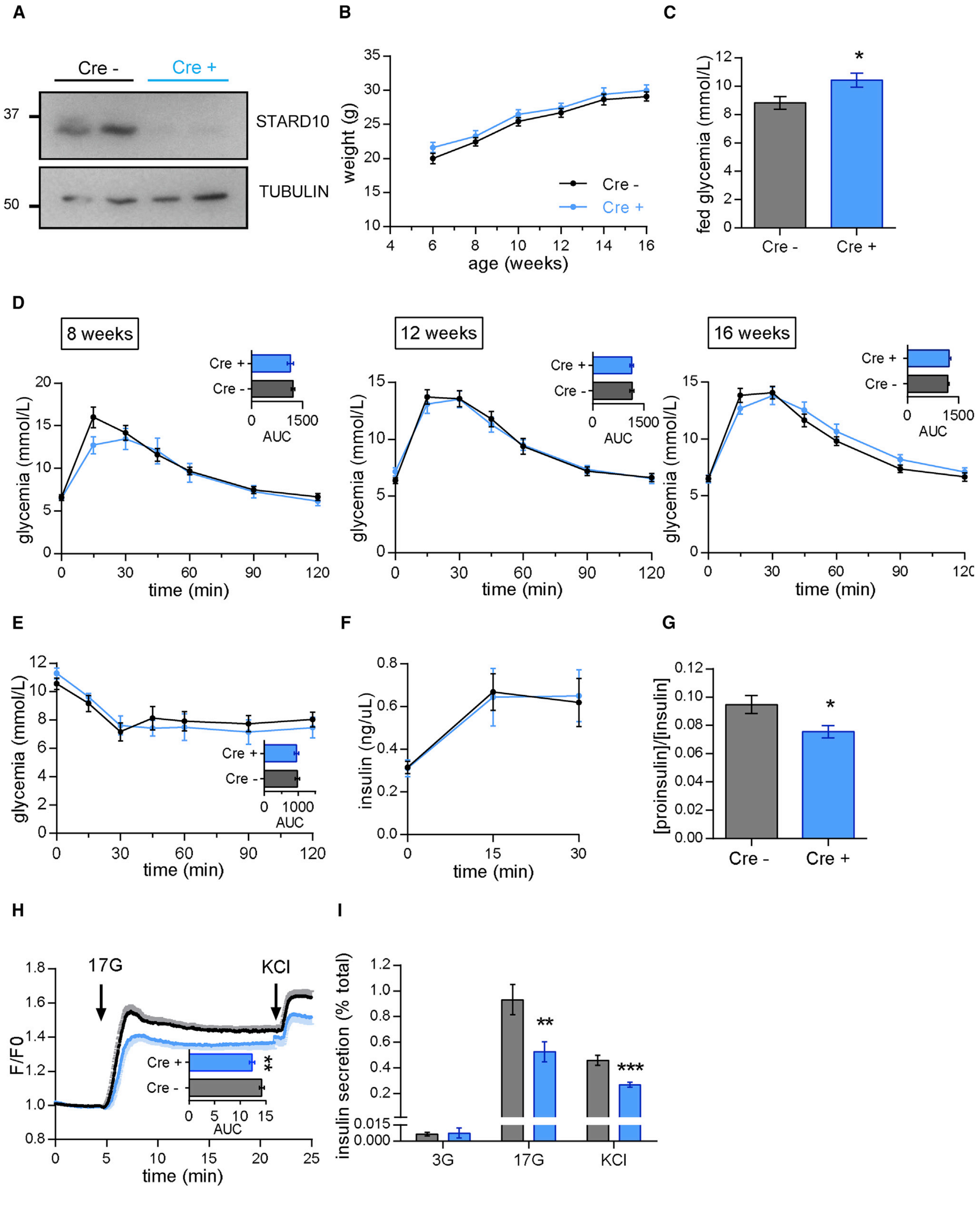

Figure 3. Defective Proinsulin Processing and In Vitro Insulin Secretion in β*StarD10* KO Mice
(A) Western blot analysis of STARD10 in pancreatic islets of wild-type (Cre^{-}) and littermate β*StarD10* KO mice (Cre^{+}).
(B) Growth curves are similar in male WT and β*StarD10* KO littermates maintained on a regular chow diet.
(C) Increased fed glycemia in 14-week-old male β*StarD10* KO compared to WT littermates (n = 8–9 mice per genotype, *$p < 0.05$, unpaired two-tailed Student's t test).
(D) Glucose tolerance at 8, 12, and 16 weeks of age are similar in male WT and β*StarD10* KO littermates as determined by intraperitoneal glucose tolerance tests (1 g/kg).

(legend continued on next page)

ABOS ("Atlas Biologique de l'Obésité Sévère") cohort (ClinicalTrials: NCT01129297)[66] collection. Since none of the variants in the credible set were detected in Metabochip samples after QC checks (see Material and Methods), we used proxy SNPs for four of the five variants (rs140130268, rs148527516, rs7103836, rs76550717). None of these proxies displayed any significant association with probes for *ARAP1* or *STARD10*.

Defective Glucose Homeostasis and Insulin Secretion in Mice Deleted Selectively for *StarD10* in the β Cell

Mice lacking *StarD10* selectively in the β cell (β*StarD10* KO) were generated by crossing *StarD10* floxed mice, in which *LoxP* sites were present at either side of exon 3, to mice carrying an Ins1*Cre* allele.[57] The latter strain allows efficient (>94%) deletion in β cells, without significant recombination at extra-pancreatic sites including the brain or the expression of human growth hormone.[67] Levels of *StarD10* mRNA (not shown) and protein (Figure 3A) were markedly reduced in islets from β*StarD10* KO mice, demonstrating efficient deletion from β cells. β*StarD10* KO mice displayed significantly higher fed glycaemia at 14 weeks of age (Figure 3C), although glucose tolerance was not different compared to control Cre^{-} littermates at 16 weeks of age (Figure 3D). Insulin sensitivity (Figure 3E) and in vivo insulin secretion in response to glucose (Figure 3F) remained unchanged in β*StarD10* KO mice versus controls. While vesicle density at the plasma membrane, assessed using total internal reflection of fluorescence (TIRF) microscopy, was not altered by *StarD10* deletion (Figures S3A and S3B), increases in the number of exocytotic events in response to glucose or KCl tended to be reduced (Figure S3C). Importantly, proinsulin:insulin ratios were significantly lower in β*StarD10* KO mice compared to controls (Figure 3G).

Indicating defects in both glucose sensing and metabolism-independent insulin secretion, cytosolic Ca^{2+} increases in response to high glucose were diminished (Figure 3H), and both glucose- and KCl-induced insulin secretion (Figure 3I) were impaired in islets from β*StarD10* KO animals, compared to controls.

Impaired Glucose Homeostasis in Global *StarD10*-Null Mice

Given the mild glycaemic defects observed in β*StarD10* KO animals and the fact that *StarD10* is highly expressed in the liver (bioGps, Web Resources), we next explored glucose homeostasis in animals deleted globally ("tm1a allele")[56] (IMPC, Web Resources) for *StarD10*. This approach generates a null allele through splicing to a *lacZ* trapping element inserted into the second intron of *StarD10*. Correspondingly, *StarD10* mRNA was eliminated from both the liver and pancreatic islets (data not shown) and STARD10 depletion from islets (Figure 4A) and liver (not shown) was verified by western blotting. Body weights of animals fed a normal chow diet were indistinguishable between genotypes (Figure 4B). However, compared to wild-type controls, male $StarD10^{+/-}$ and $StarD10^{-/-}$ mice exhibited higher fed glycemia from 14 weeks (Figure 4C) and developed glucose intolerance from 16 weeks of age (Figure 4D). $StarD10^{+/-}$ and $StarD10^{-/-}$ mice also showed markedly reduced insulin sensitivity (Figure 4E) and insulin secretion in response to glucose was sharply reduced in vivo (Figure 4F). In common with β*StarD10* KO mice, the ratio of circulating proinsulin:insulin was also diminished in $StarD10^{-/-}$ mice versus controls (Figure 4G).

Examined in isolated islets from male and female mice combined, cytosolic Ca^{2+} responses to glucose, though not KCl, were also decreased in *StarD10*-null animals, compared to wild-type mice (Figure 4H). Correspondingly, insulin secretion (Figure 4I) was markedly impaired in islets from $StarD10^{-/-}$ versus wild-type mice. KCl-stimulated insulin secretion was also strongly diminished in islets from the null mice (Figure 4I, right). By contrast, β cell mass was increased, though β:α cell ratio was not affected, in null animals (Figure S4).

Glucose Homeostasis Is Improved in Mice Overexpressing *StarD10* in Pancreatic β Cells

In light of the findings above, we generated mice in which *StarD10* was overexpressed selectively in the β cell in adults under the control of an *insulin 2* promoter-driven reverse tetracycline trans-activator (Rip7-rtTA).[55] Transgene expression was induced by doxycycline (2 g/L) at 5 weeks of age (Figure S5A). Transgenic animals fed a normal chow diet displayed no alterations in body weight (Figure S5B, solid lines) and exhibited similar glucose tolerance compared to wild-type littermates (Figure S5C). However, *StarD10*-tg male mice fed a high fat diet (HFD, 60% total calories from fat) (Figure S5C, dotted lines) displayed improved intraperitoneal glucose tolerance (16 weeks, AUC: WT: 2,586 ± 153 M.min; *StarD10*-tg: 1,818 ± 129 M.min; $p < 0.05$). These animals gained less

(E) Insulin sensitivity is similar in 17-week-old WT and β*StarD10* KO littermates as assessed by intraperitoneal insulin tolerance test (0.75 U/kg insulin). n = 8–9 mice per genotype.
(F) In vivo insulin secretion measured from plasma collected after intraperitoneal glucose injection (3 g/kg) from 18-week-old male mice are unaffected. n = 4–6 mice per genotype.
(G) Fasting plasma proinsulin:insulin ratio from male and female 20-week-old mice is decreased in β*StarD10* KO compared to WT littermates (n = 16 mice per genotype; *$p < 0.05$, unpaired two-tailed Student's t test).
(H) Intracellular Ca^{2+} responses of islets isolated from male and female mice to 17 mM glucose and 20 mM KCl (n = 5 mice per genotype; **$p < 0.01$, unpaired two-tailed Student's t test).
(I) Impaired glucose (17 mM)- and KCl (30 mM)-induced insulin secretion assessed in islets isolated from male and female mice (n = 5 mice per genotype; **$p < 0.01$, ***$p < 0.001$ unpaired two-tailed Student's t test).
All data are represented as the mean ± SEM.

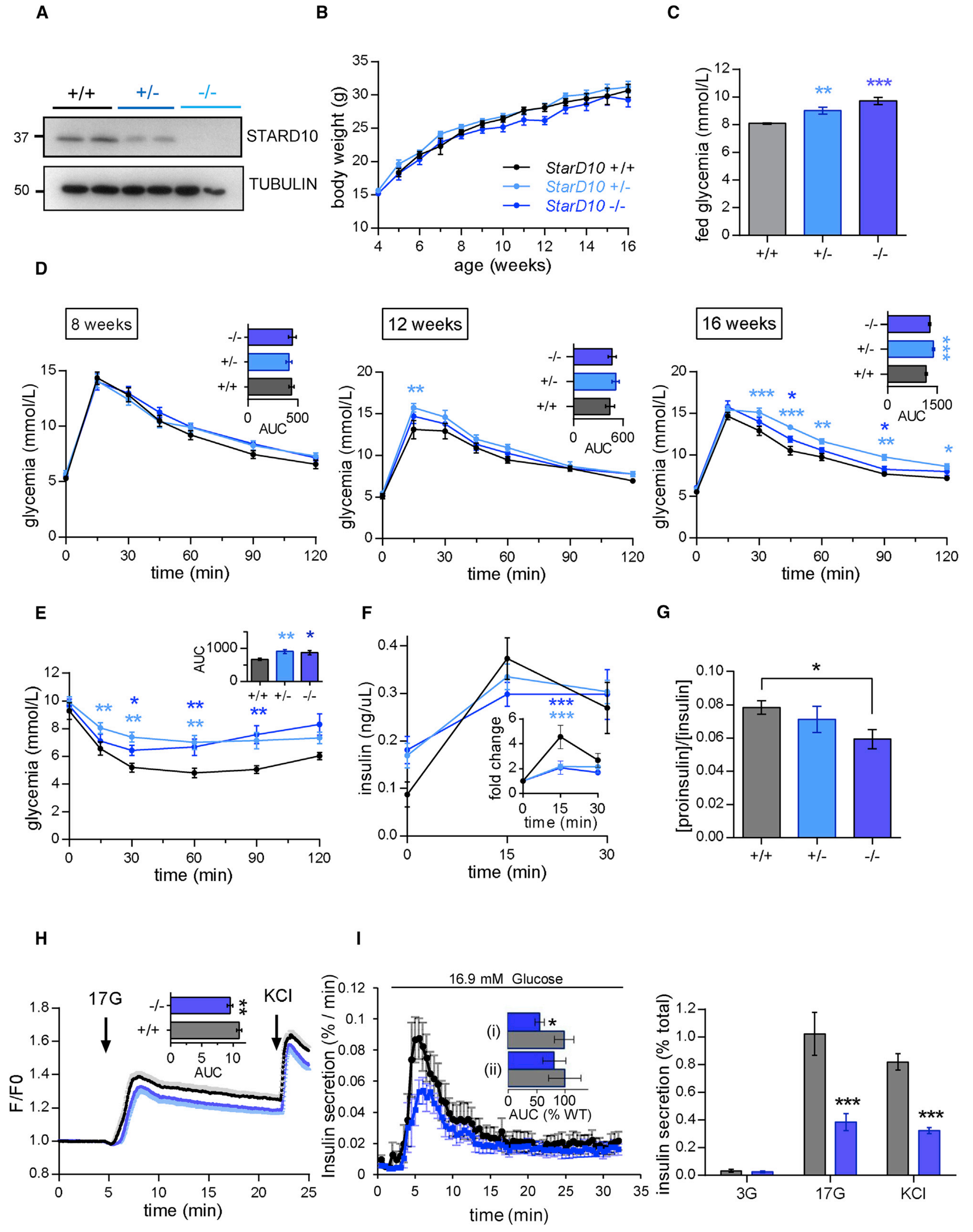

Figure 4. Defective Glucose Homeostasis, Insulin Secretion, and Proinsulin Processing in *StarD10* Global KO Mice
(A) Western blot analysis of STARD10 in pancreatic islets of $StarD10^{+/+}$, $StarD10^{+/-}$, and $StarD10^{-/-}$ mice.
(B) Growth curves are similar in *StarD10*-WT (black), heterozygous (light blue), and null (dark blue) male littermates maintained on a regular chow diet.

(legend continued on next page)

weight than control littermates (Figure S5B, dotted lines; 16 weeks, WT: 39.13 ± 1.91 g; *StarD10*-tg: 32.66 ± 1.73 g; $p < 0.01$) though insulin sensitivity was not significantly affected (Figure S5D). Glucose-induced cytosolic Ca^{2+} responses (Figure S5E) and insulin secretion (Figure S5F) were indistinguishable between isolated islets from *StarD10*-tg animals fed a normal chow diet and those isolated from wild-type littermates.

Unaltered Glucose Homeostasis and Proinsulin Processing after Deletion of *Arap1* Selectively in the Pancreatic β Cell

We next examined the impact of ablating *Arap1* expression selectively in the β cell. Mice bearing *floxed* alleles of *Arap1*, in which *LoxP* sites were present at either side of exon 12, were bred to mice carrying an Ins1*Cre* allele[57] as above. Islet ARAP1 immunoreactivity was reduced by >70%–80% (Figure 5A), reflecting selective deletion from β cells. Mice null for *Arap1* displayed normal changes in body weight (Figure 5B) and fed glycemia (Figure 5C), unaltered glucose tolerance (Figure 5D), insulin sensitivity (Figure 5E), and glucose-stimulated insulin secretion in vivo (Figure 5F). In marked contrast to βStarD10 KO mice, fasting proinsulin:insulin ratios (Figure 5G), intracellular Ca^{2+} dynamics (Figure 5H), and insulin secretion from isolated islets (Figure 5I) were indistinguishable between β*Arap1* KO mice and littermate controls.

Overexpression of *ARAP1* in Pancreatic β Cells Does Not Affect Glucose Homeostasis in Mice

Given that previous studies[31] reported an association between the possession of risk alleles and *increased* expression of *ARAP1*, we also explored the impact of overexpressing *ARAP1* in β cells (Figure S6A). There were no differences in body weight between control (black) and transgenic (red) animals (Figure S6B) fed either a regular chow (solid lines) or a high fat diet (HFD; dotted lines). Transgenic animals fed a regular chow diet (Figure S6C, solid lines) showed little evidence of abnormal glucose tolerance until 16 weeks of age, other than a small increase in peak blood glucose observed at 8 weeks of age. Similarly, transgenic animals fed a HFD (Figure S6C, dotted lines) presented with no abnormalities in intraperitoneal glucose tolerance. Intracellular free Ca^{2+} increases (Figure S6D) and insulin secretion in response to high glucose (Figure S6E) were also identical between the two genotypes.

These data further argue against a role for increased *ARAP1* expression in the β cell as responsible for the diabetogenic effects of T2D risk alleles at this locus.

Given the existence of insulin resistance in global *StarD10* mice, likely to be the result of changes in liver function, we reassessed the impact of variants near *ARAP1* and *STARD10* on this parameter in humans by consulting previously published data.[68] HOMA-IR revealed no changes in insulin sensitivity in risk allele carriers (results not shown).

Discussion

Our primary aim in the present study was to establish which gene(s) at the previously identified T2D association signal at the *ARAP1/STARD10* locus contribute to altered T2D risk in humans, and the likely tissue through which these effects were observed. Using a combination of human pancreatic islet transcriptome data, in vitro studies, and molecular genetics in mice, we provide evidence, discussed in detail below, that the pathogenic action is mediated, at least in large part, via decreases in *STARD10*, but not *ARAP1*, expression in the β cell.

First, we identify a 5 kb region in intron 2 of *STARD10* that: (1) captures the T2D GWAS association signal; (2) is associated with *STARD10*, but not *ARAP1*, mRNA expression in human islets and physically interacts with the promoter for the *STARD10* isoform dominant in islets; (3) overlays a stretch-enhancer in islets; (4) is correlated with local chromatin structure in islets; and (5) contains allelic variants shown experimentally to alter enhancer function. Though this region contains several highly correlated candidate variants, which may individually or jointly contribute to T2D risk, we demonstrate that the variant most likely to underlie the associations has a direct effect

(C) Dose-dependant increases in fed glycemia in 14-week-old male deleted for *StarD10*, compared to WT littermates (one-way ANOVA, Tukey post-test).
(D) Impaired glucose tolerance in 16-week-old male *StarD10*$^{+/-}$ and *StarD10*$^{-/-}$ compared to *StarD10*$^{+/+}$ littermates as assessed by intraperitoneal glucose tolerance (1 g/kg) (n = 8–12 mice per genotype; *$p < 0.05$, **$p < 0.01$, ***$p < 0.001$, two-way ANOVA, Sidak post-test).
(E) Intraperitoneal insulin tolerance was assessed at 17 weeks of age in mice fed a regular chow diet (0.75 U/kg insulin). Glucose excursion and area under the curve (AUC) are shown (n = 8–12 mice per genotype; *$p < 0.05$, **$p < 0.01$, ***$p < 0.001$, two-way ANOVA, Sidak post-test).
(F) In vivo insulin secretion measured from plasma collected after intraperitoneal glucose injection (3 g/kg) from 18-week-old male mice, represented in ng/mL or as fold change over basal (inset) (n = 7–8 mice per genotype; ***$p < 0.001$, two-way ANOVA, Sidak post-test).
(G) Decreased fasting plasma proinsulin:insulin ratio in 20-week-old male and female *StarD10*$^{-/-}$ versus *StarD10*$^{+/+}$ littermates (n = 10–17 mice per genotype, unpaired two-tailed Student's t test).
(H) Impaired glucose (17 mM) induced calcium responses in male and female *StarD10*$^{-/-}$ versus *StarD10*$^{+/+}$ islets (n = 4 mice per genotype; **$p < 0.01$, unpaired two-tailed Student's t test).
(I) Impaired glucose (17 mM) and KCl (30 mM) insulin secretion assessed in islets isolated from male and female β*StarD10* KO versus WT littermates, in perifusion (left; (i), 4–6.5 min; (ii), 15–32 min) or static incubation (right) (n = 4 mice per genotype, *$p < 0.05$, **$p < 0.01$, ***$p < 0.001$, unpaired two-tailed Student's t tests).
All data are represented as the mean ± SEM.

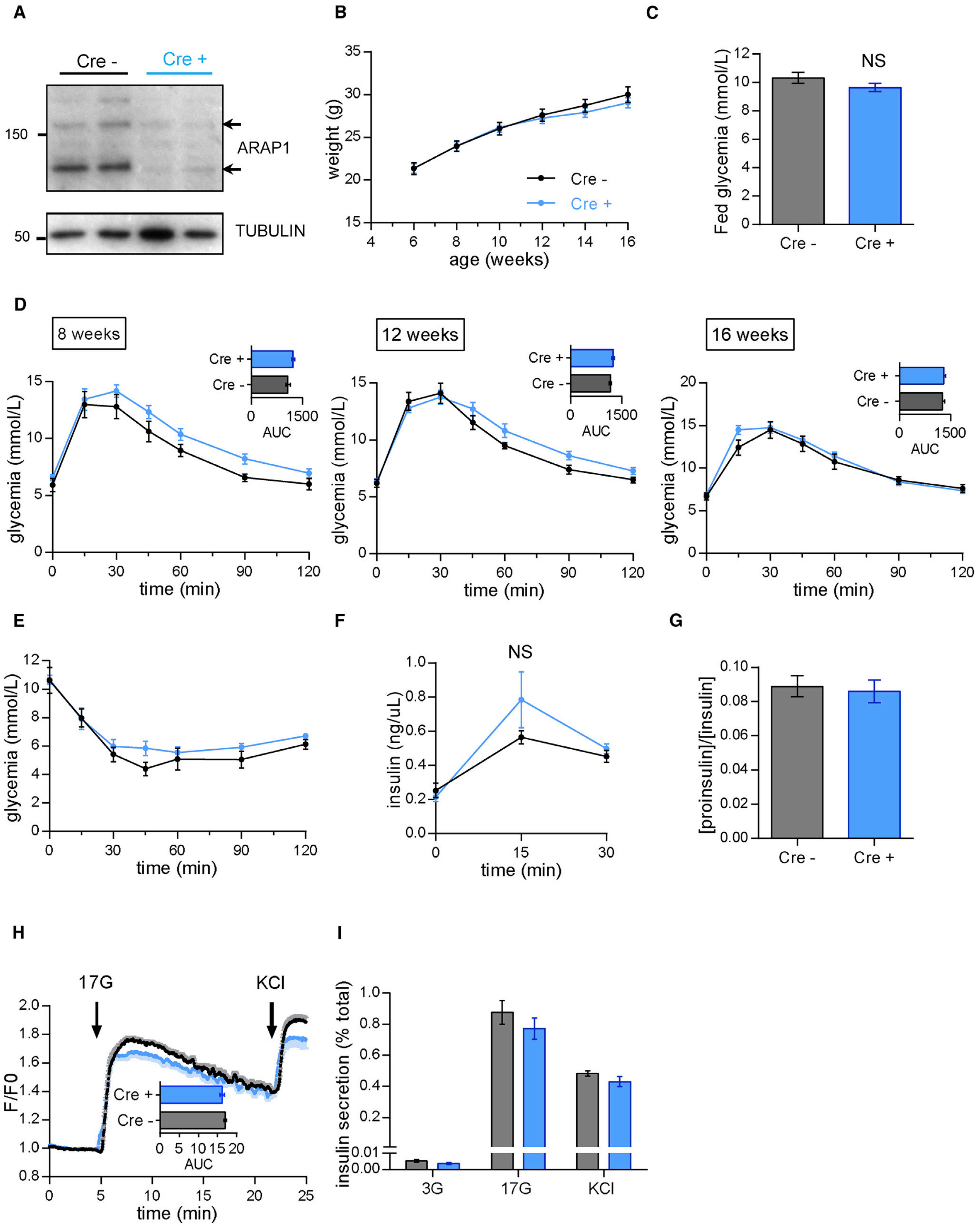

Figure 5. Normal Glucose Homeostasis, Insulin Secretion, and Proinsulin Processing in β*Arap1* KO Mice
(A) Western blot analysis of ARAP1 in pancreatic islets of WT (Cre^-) and *Arap1* β cell KO mice (Cre^+). The arrows depict the short (130 kDa) and long (160 kDa) variants of ARAP1.
(B) Growth curves are similar in male WT and β*Arap1* KO littermates maintained on a regular chow diet.
(C) Fed glycemia are identical in 14-week-old male WT and β*Arap1* KO littermates.

(legend continued on next page)

on local enhancer function. Of note, the risk allele of indel rs140130268, a gain of a GTTT repeat, is associated with lowered enhancer activity (Figure 2B). Such repeats are associated with Crohn disease[69] and neurodegenerative disorders,[70,71] in each case decreasing the transcription of nearby genes.

In contrast to recent findings,[31] but in line with other studies,[30,72] we obtained no evidence of an association between the risk allele at rs1552224 and increased *ARAP1* expression levels in islets. On the other hand, when we extended a previous study of human pancreatic islet expression[30] that had associated increased *STARD10* expression with the minor (T2D-protective) allele at the GWAS proxy variant rs1552224 and added human islet data from two new independent subject groups provided by the IMIDIA consortium, derived from both organ donors and partial pancreatectomy patients, we could show reproducible association with *STARD10* expression. The latter samples provide data for the first time from T2D subjects: interestingly, both *STARD10* and *ARAP1* expression were decreased in T2D islets versus non-diabetic controls, the former in common with earlier findings,[73] and further suggesting that STARD10 depletion may impair insulin production in the diseased state. Given the absence of a *cis*-eQTL for *ARAP1*, we interpret the reduced expression of this gene in T2D versus non-diabetic islets as being reactive rather than causal.

Although the more marked glycemic phenotype of global versus β cell-selective *StarD10* mice might appear to suggest an action of T2D-associated variants at the *ARAP1/STARD10* locus via the liver or other insulin-sensitive tissues, several lines of evidence point away from this possibility. First, changes in fed glycemia were similar in both global and β cell-selective *StarD10*-null mice and the lowering of plasma proinsulin:insulin ratio, which characterizes carriers of the human risk alleles at this locus,[19] was similarly recapitulated in both models. The latter observation strongly suggests that alterations in proinsulin processing in the β cell, rather than preferential clearance of proinsulin by the liver in the absence of *StarD10* in the latter tissue, are responsible for the altered circulating levels of the two forms of insulin (Figures 3 and 4). Deletion of *StarD10* selectively in liver cells will be needed in the future to confirm or refute this point. Second, previous GWASs and meta-analyses in man[19,20,68] demonstrated that risk variants at this locus are associated with a negative log HOMA-B, implying decreased β cell function. By contrast, no associations were found with HOMA-IR, thus indicating unaltered insulin sensitivity. In line with this, decreased insulin secretion during OGTT was observed in non-diabetic carriers of risk alleles,[21,74] with no alteration of the insulin sensitivity index. Third, a change in *STARD10* (but not *ARAP1*) expression associated with genotype was clearly observed in islets as well as from pancreatic tissue obtained by LCM and thus partially enriched in β cells (Table 1). Fourth, analysis of active histone marks and DNAase hypersensitivity reveals that the implicated variants at this locus reside in large stretch-enhancer specific to islets but largely absent from liver-derived cells (Figures 1C and S1). Fifth, the identified variants were located in a region shown by 3C analysis to be physically associated with the *STARD10* promoter in a human β cell-derived line (Figure 2A). Interestingly, a physical association was also detected between the *ARAP1* promoter and the same region (Figure S2), though the functional significance of this is obscure. Thus, sixth, eQTLs in significant LD with the T2D locus are not detected in human liver samples from the GTEx[64] or STARNET[65] consortia as well as samples from the ABOS consortium.[66] Based on the power calculation provided by the GTEx Consortium,[75] and given that the minor allele frequency for variants at the *STARD10/ARAP1* locus is 15%,[19] then with a sample size of n = 186 (ABOS)[66] or n = 600 (STARNET,)[65] we would expect a power of 90% or 100%, respectively, to detect a β of 0.15 at α 2.5×10^{-7}. In neither of these cases, nor in the GTex samples (n = 97),[64] are liver *cis*-eQTLs detected for either *STARD10* or *ARAP1*. Although the above power calculations[75] are somewhat imprecise given differing expression levels, the latter two studies[65,66] are thus adequately powered to detect *cis*-eQTLs for *STARD10* or *ARAP1* in the liver of the same size or smaller than that seen in islets. Instead, our analysis of more than 800 liver samples from three separate groups fails to identify any liver *cis*-eQTLs for either *ARAP1* or *STARD10* of comparable size to that detected for *STARD10* in islet samples from 341 subjects (Figure 1, Table 1). Nevertheless, we do not exclude the possibility that future, even larger eQTL studies, might identify more subtle effects on the expression of additional genes at this locus.

Studies in mice also provided further functional evidence for *STARD10* as the gene most likely to confer effects

(D) Unaffected glucose tolerance in 8-, 12-, and 16-week-old male β*Arap1* KO versus WT littermates as determined by intraperitoneal glucose tolerance tests (1 g/kg).
(E) Insulin sensitivity is similar in 17-week-old male WT and β*Arap1* KO littermates as assessed by intraperitoneal insulin tolerance test (0.75 U/kg insulin). n = 8–14 mice per genotype.
(F) In vivo insulin secretion measured from plasma collected after intraperitoneal glucose injection (3 g/kg) from 18-week-old male mice remain unaffected. n = 7–10 mice per genotype.
(G) Fasting plasma proinsulin:insulin ratios are similar in 20-week-old male and female WT and β*Arap1* KO littermates; n = 16–23 mice per genotype.
(H) Calcium responses of isolated islets to 17 mM glucose and 20 mM KCl are unaffected.
(I) Insulin secretion assessed in isolated islets remain similar between genotypes; n = 3–7 mice per genotype.
All data are represented as the mean ± SEM.

on T2D risk at this locus. Thus, highly targeted disruption of *Arap1* in β cells had no effect on insulin secretion in vivo or in vitro nor on circulating proinsulin:insulin ratios. Furthermore, inducible overexpression of *ARAP1* in β cells in adult mice failed to exert substantial effects on insulin secretion or glucose tolerance. Again, global or liver-specific deletion of *Arap1* will be useful in the future to exclude any possible contribution of this gene to the action of risk variants through the latter tissue.

Together, this body of evidence points toward an action of the T2D-associated variants via the β cell and through changes in *STARD10* expression. Futher work will need to be undertaken to explore the molecular mechanisms through which the observed variants affect *STARD10* expression, including the identification of transcription factors which bind in this region. While interrogation of the Islet Regulome browser (Web Resources)[34] reveals that none of five key β cell transcription factors examined by ChIP-seq in human islets (FOXA2, MAFB, NKX2.2, NKX6.1, and PDX1) bind at this site, the binding of High Mobility Group Box 1 (HMGB1) is predicted to be affected by GTTT deletion.[76] However, our preliminary experiments (not shown) have failed to provide any evidence to support this possibility.

We would note that the relevance of changes in insulin sensitivity in *StarD10*-null mice as regards impact of T2D variants at the *ARAP1/STARD10* locus is questionable, given the absence of any evidence for an impact of this locus on insulin signaling (see above). Nevertheless, the more striking glycemic phenotype of the global versus the β cell-selective *StarD10*-null mouse (Figure 3 versus Figure 4) means that the extra-pancreatic actions of therapeutic agents that seek to target STARD10 will need to be given careful consideration.

Possible Mechanisms of STARD10 Action on Insulin Processing and Secretion

By what means may STARD10 depletion impair insulin secretion while preserving proinsulin processing? First, since STARD10 is concentrated in sperm flagella,[26] a site of vigorous energy comsumption, the enzyme might conceivably be required for normal glucose metabolism and signaling in the β cells.[77] Supporting this view, glucose-induced Ca^{2+} dynamics, likely reflecting glucose-dependent ATP generation, were impaired in *StarD10*-null β cells. On the other hand, preliminary lipidomic analysis in liver (not shown) indicates changes that, if they also affect the β cell granule or plasma membranes, may impair exocytosis or favor intracellular retention of unprocessed insulin. Finally, impaired exocytosis may of itself improve processing by increasing dwell time in the maturing granule.[19] Interestingly, we saw no significant change in the number of morphologically docked granules at the plasma membrane by TIRF imaging (Figure S3), despite a trend ($p = 0.06$) toward a reduction in secretion in response to depolarization with KCl, implying impaired exocytotic competence of granules. This may be consistent with altered granule membrane lipid composition and defective incorporation of SNAP/SNARE proteins.[78] Finally, transcriptional mechanisms, as proposed for the control by STARD10 of PPARα (MIM: 170998),[29] may also play a role.

Conclusions

The present study has used multiple complementary approaches to assess the identity of the gene(s) and the site(s) of action of variants at a locus on chromosome 11q associated with T2D risk in several earlier studies.[19–22] This multi-faceted approach has been adopted since, in our view, no one single piece of evidence can be considered definitive in connecting true GWAS signals to their downstream effectors as each has intrinsic limitations. Rather, it is the alignment of multiple types of data, each supporting the same hypothesis which, as here, provides compelling evidence to establish a particular gene as the effector transcript at a given locus.

Although it is anticipated that future studies will bring important additional insights, our findings challenge the existing view that alterations in *ARAP1* expression in the β cell represent the sole or most important mechanism[31] but instead imply a role for *STARD10* in this tissue. The molecular mechanisms through which the encoded lipid transfer protein affects β cell physiology, and in particular proinsulin processing, should provide exciting avenues for future research and possibly therapeutic exploitation.

Accession Numbers

Raw sequence files for the ATAC-seq data described in this manuscript are available from the European Nucleotide Archive (ENA) under accession number PRJEB18684.

Supplemental Data

Supplemental Data include six figures and one table and can be found with this article online at http://dx.doi.org/10.1016/j.ajhg.2017.01.011.

Acknowledgments

G.A.R. was supported by a Wellcome Trust Senior Investigator Award (WT098424AIA), an MRC Programme (MR/J0003042/1), Biotechnology and Biological Sciences Research Council (BB/J015873/1), Diabetes UK (BDA/11/0004210, BDA/15/0005275), Imperial Confidence in Concept (ICiC) grants, and a Royal Society Wolfson Research Merit Award. G.R.C. received a Fellowship from the Societé Francophone du Diabète. This work was also supported by an MRC Experimental Challenge grant (MR/L020149/1) to the DIVA consortium and by the Innovative Medicines Initiative Joint Undertaking under grant agreement 155005 (IMIDIA), resources of which are composed of financial contribution from the European Union's Seventh Framework Programme (FP7/2007-2013) and European Federation of Pharmaceutical Industries and Associations (EFPIA) companies. We thank Raphael Scharfmann (Inserm U1016, Université Paris Descartes, Paris, France) for advice on the use of EndoC-βH1 cells and Professor Timothy Frayling

(University of Exeter) for re-analysis of data from the MAGIC consortium. A.L.G. is a Wellcome Trust Senior Fellow in Basic Biomedical Science (095101/Z/10/Z). M.I.M. is a Wellcome Trust Senior Investigator and received Wellcome Trust grants 090532, 098381, and 106130, NIH (US) awards UO1-DK105535 and RO1-MH101814, MRC Programme MR/L020149/1, and funds from the Oxford NIHR Biomedical Research Centre. M.v.d.B. is supported by a Novo Nordisk postdoctoral fellowship run in partnership with the University of Oxford. Human islet isolation in Edmonton was funded in part by the Alberta Diabetes Foundation and the University of Alberta. P.E.M. was supported by a 2016-2017 Killam Annual Professorship. K.J.G. is supported by NIH grant P30-DK063491.

Received: July 25, 2016
Accepted: December 20, 2016
Published: January 26, 2017

Web Resources

BioGPS, http://biogps.org/
ClinicalTrials.gov, http://clinicaltrials.gov
DNase and ChIP pipeline, http://userweb.molbiol.ox.ac.uk/public/telenius/PipeSite.html
European Nucleotide Archive, http://www.ebi.ac.uk/ena
FastQC, http://www.bioinformatics.babraham.ac.uk/projects/fastqc
IMIDIA, http://www.imidia.org
International Mouse Phenotyping Consortium, http://www.mousephenotype.org/
Islet regulome, http://gattaca.imppc.org/isletregulome/home
Knockout Mouse Project (KOMP) Repository, https://www.komp.org/
OMIM, http://www.omim.org/
PLINK, http://pngu.mgh.harvard.edu/~purcell/plink/
Trim Galore, http://www.bioinformatics.babraham.ac.uk/projects/trim_galore/

References

1. Steiner, D.F. (1990). The biosynthesis of insulin. In Insulin, P. Cuatracasas and S. Jacobs, eds. (Berlin, Heidelberg: Springer-Verlag), pp. 67–92.
2. Thorens, B., Sarkar, H.K., Kaback, H.R., and Lodish, H.F. (1988). Cloning and functional expression in bacteria of a novel glucose transporter present in liver, intestine, kidney, and beta-pancreatic islet cells. Cell *55*, 281–290.
3. Matschinsky, F.M., and Collins, H.W. (1997). Essential biochemical design features of the fuel-sensing system in pancreatic beta-cells. Chem. Biol. *4*, 249–257.
4. Rutter, G.A. (2004). Visualising insulin secretion. The Minkowski Lecture 2004. Diabetologia *47*, 1861–1872.
5. Tarasov, A.I., Griffiths, E.J., and Rutter, G.A. (2012). Regulation of ATP production by mitochondrial Ca(2+). Cell Calcium *52*, 28–35.
6. Ashcroft, F.M., and Rorsman, P. (2013). K(ATP) channels and islet hormone secretion: new insights and controversies. Nat. Rev. Endocrinol. *9*, 660–669.
7. Rutter, G.A., Theler, J.-M., Murgia, M., Wollheim, C.B., Pozzan, T., and Rizzuto, R. (1993). Stimulated Ca2+ influx raises mitochondrial free Ca2+ to supramicromolar levels in a pancreatic β-cell line. Possible role in glucose and agonist-induced insulin secretion. J. Biol. Chem. *268*, 22385–22390.
8. Henquin, J.C. (2000). Triggering and amplifying pathways of regulation of insulin secretion by glucose. Diabetes *49*, 1751–1760.
9. Rutter, G.A., Pullen, T.J., Hodson, D.J., and Martinez-Sanchez, A. (2015). Pancreatic β-cell identity, glucose sensing and the control of insulin secretion. Biochem. J. *466*, 203–218.
10. Butler, A.E., Janson, J., Bonner-Weir, S., Ritzel, R., Rizza, R.A., and Butler, P.C. (2003). Beta-cell deficit and increased beta-cell apoptosis in humans with type 2 diabetes. Diabetes *52*, 102–110.
11. Del Guerra, S., Lupi, R., Marselli, L., Masini, M., Bugliani, M., Sbrana, S., Torri, S., Pollera, M., Boggi, U., Mosca, F., et al. (2005). Functional and molecular defects of pancreatic islets in human type 2 diabetes. Diabetes *54*, 727–735.
12. Kahn, S.E. (2003). The relative contributions of insulin resistance and beta-cell dysfunction to the pathophysiology of type 2 diabetes. Diabetologia *46*, 3–19.
13. Rutter, G.A., and Parton, L.E. (2008). The beta-cell in type 2 diabetes and in obesity. Front. Horm. Res. *36*, 118–134.
14. Marullo, L., El-Sayed Moustafa, J.S., and Prokopenko, I. (2014). Insights into the genetic susceptibility to type 2 diabetes from genome-wide association studies of glycaemic traits. Curr. Diab. Rep. *14*, 551.
15. Mohlke, K.L., and Boehnke, M. (2015). Recent advances in understanding the genetic architecture of type 2 diabetes. Hum. Mol. Genet. *24* (R1), R85–R92.
16. Rutter, G.A. (2014). Dorothy Hodgkin Lecture 2014: understanding GWAS genes for type 2 diabetes. Diabet. Med. *31*, 1480–1487.
17. Nolan, C.J., and Delghingaro-Augusto, V. (2016). Reversibility of defects in proinsulin processing and islet β-cell failure in obesity-related type 2 diabetes. Diabetes *65*, 352–354.
18. Halban, P.A., Polonsky, K.S., Bowden, D.W., Hawkins, M.A., Ling, C., Mather, K.J., Powers, A.C., Rhodes, C.J., Sussel, L., and Weir, G.C. (2014). β-cell failure in type 2 diabetes: postulated mechanisms and prospects for prevention and treatment. Diabetes Care *37*, 1751–1758.
19. Strawbridge, R.J., Dupuis, J., Prokopenko, I., Barker, A., Ahlqvist, E., Rybin, D., Petrie, J.R., Travers, M.E., Bouatia-Naji, N., Dimas, A.S., et al.; DIAGRAM Consortium; GIANT Consortium; MuTHER Consortium; CARDIoGRAM Consortium; and C4D Consortium (2011). Genome-wide association identifies nine common variants associated with fasting proinsulin levels and provides new insights into the pathophysiology of type 2 diabetes. Diabetes *60*, 2624–2634.
20. Voight, B.F., Scott, L.J., Steinthorsdottir, V., Morris, A.P., Dina, C., Welch, R.P., Zeggini, E., Huth, C., Aulchenko, Y.S., Thorleifsson, G., et al.; MAGIC investigators; and GIANT Consortium (2010). Twelve type 2 diabetes susceptibility loci identified through large-scale association analysis. Nat. Genet. *42*, 579–589.
21. Nielsen, T., Sparsø, T., Grarup, N., Jørgensen, T., Pisinger, C., Witte, D.R., Hansen, T., Pedersen, O.; and Diabetes Genetics Replication and Meta-analysis (DIAGRAM) Consortium (2011). Type 2 diabetes risk allele near CENTD2 is associated with decreased glucose-stimulated insulin release. Diabetologia *54*, 1052–1056.
22. Dimas, A.S., Lagou, V., Barker, A., Knowles, J.W., Mägi, R., Hivert, M.F., Benazzo, A., Rybin, D., Jackson, A.U., Stringham, H.M., et al.; MAGIC Investigators (2014). Impact of type 2

diabetes susceptibility variants on quantitative glycemic traits reveals mechanistic heterogeneity. Diabetes *63*, 2158–2171.
23. Cuthbert, E.J., Davis, K.K., and Casanova, J.E. (2008). Substrate specificities and activities of AZAP family Arf GAPs in vivo. Am. J. Physiol. Cell Physiol. *294*, C263–C270.
24. Lawrence, J.T., and Birnbaum, M.J. (2003). ADP-ribosylation factor 6 regulates insulin secretion through plasma membrane phosphatidylinositol 4,5-bisphosphate. Proc. Natl. Acad. Sci. USA *100*, 13320–13325.
25. Olayioye, M.A., Vehring, S., Müller, P., Herrmann, A., Schiller, J., Thiele, C., Lindeman, G.J., Visvader, J.E., and Pomorski, T. (2005). StarD10, a START domain protein overexpressed in breast cancer, functions as a phospholipid transfer protein. J. Biol. Chem. *280*, 27436–27442.
26. Yamanaka, M., Koga, M., Tanaka, H., Nakamura, Y., Ohta, H., Yomogida, K., Tsuchida, J., Iguchi, N., Nojima, H., Nozaki, M., et al. (2000). Molecular cloning and characterization of phosphatidylcholine transfer protein-like protein gene expressed in murine haploid germ cells. Biol. Reprod. *62*, 1694–1701.
27. Kone, M., Pullen, T.J., Sun, G., Ibberson, M., Martinez-Sanchez, A., Sayers, S., Nguyen-Tu, M.S., Kantor, C., Swisa, A., Dor, Y., et al. (2014). LKB1 and AMPK differentially regulate pancreatic β-cell identity. FASEB J. *28*, 4972–4985.
28. Blodgett, D.M., Nowosielska, A., Afik, S., Pechhold, S., Cura, A.J., Kennedy, N.J., Kim, S., Kucukural, A., Davis, R.J., Kent, S.C., et al. (2015). Novel observations from next-generation RNA sequencing of highly purified human adult and fetal islet cell subsets. Diabetes *64*, 3172–3181.
29. Ito, M., Yamanashi, Y., Toyoda, Y., Izumi-Nakaseko, H., Oda, S., Sugiyama, A., Kuroda, M., Suzuki, H., Takada, T., and Adachi-Akahane, S. (2013). Disruption of Stard10 gene alters the PPARα-mediated bile acid homeostasis. Biochim. Biophys. Acta *1831*, 459–468.
30. van de Bunt, M., Manning Fox, J.E., Dai, X., Barrett, A., Grey, C., Li, L., Bennett, A.J., Johnson, P.R., Rajotte, R.V., Gaulton, K.J., et al. (2015). Transcript expression data from human islets links regulatory signals from genome-wide association studies for type 2 diabetes and glycemic traits to their downstream effectors. PLoS Genet. *11*, e1005694.
31. Kulzer, J.R., Stitzel, M.L., Morken, M.A., Huyghe, J.R., Fuchsberger, C., Kuusisto, J., Laakso, M., Boehnke, M., Collins, F.S., and Mohlke, K.L. (2014). A common functional regulatory variant at a type 2 diabetes locus upregulates ARAP1 expression in the pancreatic beta cell. Am. J. Hum. Genet. *94*, 186–197.
32. Gaulton, K.J., Ferreira, T., Lee, Y., Raimondo, A., Mägi, R., Reschen, M.E., Mahajan, A., Locke, A., Rayner, N.W., Robertson, N., et al.; DIAbetes Genetics Replication And Meta-analysis (DIAGRAM) Consortium (2015). Genetic fine mapping and genomic annotation defines causal mechanisms at type 2 diabetes susceptibility loci. Nat. Genet. *47*, 1415–1425.
33. Wakefield, J. (2007). A Bayesian measure of the probability of false discovery in genetic epidemiology studies. Am. J. Hum. Genet. *81*, 208–227.
34. Pasquali, L., Gaulton, K.J., Rodríguez-Seguí, S.A., Mularoni, L., Miguel-Escalada, I., Akerman, I., Tena, J.J., Morán, I., Gómez-Marín, C., van de Bunt, M., et al. (2014). Pancreatic islet enhancer clusters enriched in type 2 diabetes risk-associated variants. Nat. Genet. *46*, 136–143.
35. Parker, S.C., Stitzel, M.L., Taylor, D.L., Orozco, J.M., Erdos, M.R., Akiyama, J.A., van Bueren, K.L., Chines, P.S., Narisu, N., Black, B.L., et al.; NISC Comparative Sequencing Program; National Institutes of Health Intramural Sequencing Center Comparative Sequencing Program Authors; and NISC Comparative Sequencing Program Authors (2013). Chromatin stretch enhancer states drive cell-specific gene regulation and harbor human disease risk variants. Proc. Natl. Acad. Sci. USA *110*, 17921–17926.
36. Trynka, G., Sandor, C., Han, B., Xu, H., Stranger, B.E., Liu, X.S., and Raychaudhuri, S. (2013). Chromatin marks identify critical cell types for fine mapping complex trait variants. Nat. Genet. *45*, 124–130.
37. Fuchsberger, C., Flannick, J., Teslovich, T.M., Mahajan, A., Agarwala, V., Gaulton, K.J., Ma, C., Fontanillas, P., Moutsianas, L., McCarthy, D.J., et al. (2016). The genetic architecture of type 2 diabetes. Nature *536*, 41–47.
38. Mikkelsen, T.S., Xu, Z., Zhang, X., Wang, L., Gimble, J.M., Lander, E.S., and Rosen, E.D. (2010). Comparative epigenomic analysis of murine and human adipogenesis. Cell *143*, 156–169.
39. ENCODE Project Consortium (2012). An integrated encyclopedia of DNA elements in the human genome. Nature *489*, 57–74.
40. Pickrell, J.K. (2014). Joint analysis of functional genomic data and genome-wide association studies of 18 human traits. Am. J. Hum. Genet. *94*, 559–573.
41. Buenrostro, J.D., Giresi, P.G., Zaba, L.C., Chang, H.Y., and Greenleaf, W.J. (2013). Transposition of native chromatin for fast and sensitive epigenomic profiling of open chromatin, DNA-binding proteins and nucleosome position. Nat. Methods *10*, 1213–1218.
42. Hay, D., Hughes, J.R., Babbs, C., Davies, J.O.J., Graham, B.J., Hanssen, L.L., Kassouf, M.T., Oudelaar, A.M., Sharpe, J.A., Suciu, M.C., et al. (2016). Genetic dissection of the α-globin super-enhancer in vivo. Nat. Genet. *48*, 895–903.
43. Langmead, B., Trapnell, C., Pop, M., and Salzberg, S.L. (2009). Ultrafast and memory-efficient alignment of short DNA sequences to the human genome. Genome Biol. *10*, R25–R10.
44. Magoč, T., and Salzberg, S.L. (2011). FLASH: fast length adjustment of short reads to improve genome assemblies. Bioinformatics *27*, 2957–2963.
45. van de Geijn, B., McVicker, G., Gilad, Y., and Pritchard, J.K. (2015). WASP: allele-specific software for robust molecular quantitative trait locus discovery. Nat. Methods *12*, 1061–1063.
46. Purcell, S., Neale, B., Todd-Brown, K., Thomas, L., Ferreira, M.A., Bender, D., Maller, J., Sklar, P., de Bakker, P.I., Daly, M.J., and Sham, P.C. (2007). PLINK: a tool set for whole-genome association and population-based linkage analyses. Am. J. Hum. Genet. *81*, 559–575.
47. Shabalin, A.A. (2012). Matrix eQTL: ultra fast eQTL analysis via large matrix operations. Bioinformatics *28*, 1353–1358.
48. Delaneau, O., Marchini, J.; 1000 Genomes Project Consortium; and 1000 Genomes Project Consortium (2014). Integrating sequence and array data to create an improved 1000 Genomes Project haplotype reference panel. Nat. Commun. *5*, 3934.
49. Howie, B.N., Donnelly, P., and Marchini, J. (2009). A flexible and accurate genotype imputation method for the next generation of genome-wide association studies. PLoS Genet. *5*, e1000529.
50. Dobin, A., Davis, C.A., Schlesinger, F., Drenkow, J., Zaleski, C., Jha, S., Batut, P., Chaisson, M., and Gingeras, T.R. (2013). STAR: ultrafast universal RNA-seq aligner. Bioinformatics *29*, 15–21.

51. Stegle, O., Parts, L., Durbin, R., and Winn, J. (2010). A Bayesian framework to account for complex non-genetic factors in gene expression levels greatly increases power in eQTL studies. PLoS Comput. Biol. *6*, e1000770.
52. Ongen, H., Buil, A., Brown, A.A., Dermitzakis, E.T., and Delaneau, O. (2016). Fast and efficient QTL mapper for thousands of molecular phenotypes. Bioinformatics *32*, 1479–1485.
53. Voight, B.F., Kang, H.M., Ding, J., Palmer, C.D., Sidore, C., Chines, P.S., Burtt, N.P., Fuchsberger, C., Li, Y., Erdmann, J., et al. (2012). The metabochip, a custom genotyping array for genetic studies of metabolic, cardiovascular, and anthropometric traits. PLoS Genet. *8*, e1002793.
54. Milo-Landesman, D., Surana, M., Berkovich, I., Compagni, A., Christofori, G., Fleischer, N., and Efrat, S. (2001). Correction of hyperglycemia in diabetic mice transplanted with reversibly immortalized pancreatic beta cells controlled by the tet-on regulatory system. Cell Transplant. *10*, 645–650.
55. Pullen, T.J., Sylow, L., Sun, G., Halestrap, A.P., Richter, E.A., and Rutter, G.A. (2012). Overexpression of monocarboxylate transporter-1 (SLC16A1) in mouse pancreatic β-cells leads to relative hyperinsulinism during exercise. Diabetes *61*, 1719–1725.
56. Dickinson, M.E., Flenniken, A.M., Ji, X., Teboul, L., Wong, M.D., White, J.K., Meehan, T.F., Weninger, W.J., Westerberg, H., Adissu, H., et al.; International Mouse Phenotyping Consortium; Jackson Laboratory; Infrastructure Nationale PHENOMIN, Institut Clinique de la Souris (ICS); Charles River Laboratories; MRC Harwell; Toronto Centre for Phenogenomics; Wellcome Trust Sanger Institute; and RIKEN BioResource Center (2016). High-throughput discovery of novel developmental phenotypes. Nature *537*, 508–514.
57. Thorens, B., Tarussio, D., Maestro, M.A., Rovira, M., Heikkilä, E., and Ferrer, J. (2015). Ins1(Cre) knock-in mice for beta cell-specific gene recombination. Diabetologia *58*, 558–565.
58. Truett, G.E., Heeger, P., Mynatt, R.L., Truett, A.A., Walker, J.A., and Warman, M.L. (2000). Preparation of PCR-quality mouse genomic DNA with hot sodium hydroxide and tris (HotSHOT). Biotechniques *29*, 52–54.
59. Ravier, M.A., and Rutter, G.A. (2010). Isolation and culture of mouse pancreatic islets for ex vivo imaging studies with trappable or recombinant fluorescent probes. Methods Mol. Biol. *633*, 171–184.
60. Hohmeier, H.E., Mulder, H., Chen, G., Henkel-Rieger, R., Prentki, M., and Newgard, C.B. (2000). Isolation of INS-1-derived cell lines with robust ATP-sensitive K+ channel-dependent and -independent glucose-stimulated insulin secretion. Diabetes *49*, 424–430.
61. Hagège, H., Klous, P., Braem, C., Splinter, E., Dekker, J., Cathala, G., de Laat, W., and Forné, T. (2007). Quantitative analysis of chromosome conformation capture assays (3C-qPCR). Nat. Protoc. *2*, 1722–1733.
62. Morris, A.P., Voight, B.F., Teslovich, T.M., Ferreira, T., Segrè, A.V., Steinthorsdottir, V., Strawbridge, R.J., Khan, H., Grallert, H., Mahajan, A., et al.; Wellcome Trust Case Control Consortium; Meta-Analyses of Glucose and Insulin-related traits Consortium (MAGIC) Investigators; Genetic Investigation of ANthropometric Traits (GIANT) Consortium; Asian Genetic Epidemiology Network–Type 2 Diabetes (AGEN-T2D) Consortium; South Asian Type 2 Diabetes (SAT2D) Consortium; and DIAbetes Genetics Replication And Meta-analysis (DIAGRAM) Consortium (2012). Large-scale association analysis provides insights into the genetic architecture and pathophysiology of type 2 diabetes. Nat. Genet. *44*, 981–990.
63. Ravassard, P., Hazhouz, Y., Pechberty, S., Bricout-Neveu, E., Armanet, M., Czernichow, P., and Scharfmann, R. (2011). A genetically engineered human pancreatic β cell line exhibiting glucose-inducible insulin secretion. J. Clin. Invest. *121*, 3589–3597.
64. GTEx Consortium (2015). Human genomics. The Genotype-Tissue Expression (GTEx) pilot analysis: multitissue gene regulation in humans. Science *348*, 648–660.
65. Franzén, O., Ermel, R., Cohain, A., Akers, N.K., Di Narzo, A., Talukdar, H.A., Foroughi-Asl, H., Giambartolomei, C., Fullard, J.F., Sukhavasi, K., et al. (2016). Cardiometabolic risk loci share downstream cis- and trans-gene regulation across tissues and diseases. Science *353*, 827–830.
66. Caiazzo, R., Lassailly, G., Leteurtre, E., Baud, G., Verkindt, H., Raverdy, V., Buob, D., Pigeyre, M., Mathurin, P., and Pattou, F. (2014). Roux-en-Y gastric bypass versus adjustable gastric banding to reduce nonalcoholic fatty liver disease: a 5-year controlled longitudinal study. Ann. Surg. *260*, 893–898, discussion 898–899.
67. Brouwers, B., de Faudeur, G., Osipovich, A.B., Goyvaerts, L., Lemaire, K., Boesmans, L., Cauwelier, E.J., Granvik, M., Pruniau, V.P., Van Lommel, L., et al. (2014). Impaired islet function in commonly used transgenic mouse lines due to human growth hormone minigene expression. Cell Metab. *20*, 979–990.
68. Scott, R.A., Lagou, V., Welch, R.P., Wheeler, E., Montasser, M.E., Luan, J., Mägi, R., Strawbridge, R.J., Rehnberg, E., Gustafsson, S., et al.; DIAbetes Genetics Replication and Meta-analysis (DIAGRAM) Consortium (2012). Large-scale association analyses identify new loci influencing glycemic traits and provide insight into the underlying biological pathways. Nat. Genet. *44*, 991–1005.
69. Prescott, N.J., Dominy, K.M., Kubo, M., Lewis, C.M., Fisher, S.A., Redon, R., Huang, N., Stranger, B.E., Blaszczyk, K., Hudspith, B., et al. (2010). Independent and population-specific association of risk variants at the IRGM locus with Crohn's disease. Hum. Mol. Genet. *19*, 1828–1839.
70. Rezazadeh, M., Gharesouran, J., Mirabzadeh, A., Khorram Khorshid, H.R., Biglarian, A., and Ohadi, M. (2015). A primate-specific functional GTTT-repeat in the core promoter of CYTH4 is linked to bipolar disorder in human. Prog. Neuropsychopharmacol. Biol. Psychiatry *56*, 161–167.
71. Rezazadeh, M., Gharesouran, J., Movafagh, A., Taheri, M., Darvish, H., Emamalizadeh, B., Shahmohammadibeni, N., Khorram Khorshid, H.R., Behmanesh, M., Sahraian, M.A., and Ohadi, M. (2015). Dominant and protective role of the CYTH4 primate-specific GTTT-repeat longer alleles against neurodegeneration. J. Mol. Neurosci. *56*, 593–596.
72. Locke, J.M., Hysenaj, G., Wood, A.R., Weedon, M.N., and Harries, L.W. (2015). Targeted allelic expression profiling in human islets identifies cis-regulatory effects for multiple variants identified by type 2 diabetes genome-wide association studies. Diabetes *64*, 1484–1491.
73. Fadista, J., Vikman, P., Laakso, E.O., Mollet, I.G., Esguerra, J.L., Taneera, J., Storm, P., Osmark, P., Ladenvall, C., Prasad, R.B., et al. (2014). Global genomic and transcriptomic analysis of human pancreatic islets reveals novel genes influencing glucose metabolism. Proc. Natl. Acad. Sci. USA *111*, 13924–13929.

74. Jonsson, A., Ladenvall, C., Ahluwalia, T.S., Kravic, J., Krus, U., Taneera, J., Isomaa, B., Tuomi, T., Renström, E., Groop, L., and Lyssenko, V. (2013). Effects of common genetic variants associated with type 2 diabetes and glycemic traits on α- and β-cell function and insulin action in humans. Diabetes *62*, 2978–2983.

75. GTEx Consortium (2013). The Genotype-Tissue Expression (GTEx) project. Nat. Genet. *45*, 580–585.

76. Klune, J.R., Dhupar, R., Cardinal, J., Billiar, T.R., and Tsung, A. (2008). HMGB1: endogenous danger signaling. Mol. Med. *14*, 476–484.

77. Alpy, F., and Tomasetto, C. (2005). Give lipids a START: the StAR-related lipid transfer (START) domain in mammals. J. Cell Sci. *118*, 2791–2801.

78. Rorsman, P., and Renström, E. (2003). Insulin granule dynamics in pancreatic beta cells. Diabetologia *46*, 1029–1045.

The American Journal of Human Genetics, Volume *100*

Supplemental Data

Decreased *STARD10* Expression Is Associated with Defective Insulin Secretion in Humans and Mice

Gaelle R. Carrat, Ming Hu, Marie-Sophie Nguyen-Tu, Pauline Chabosseau, Kyle J. Gaulton, Martijn van de Bunt, Afshan Siddiq, Mario Falchi, Matthias Thurner, Mickaël Canouil, Francois Pattou, Isabelle Leclerc, Timothy J. Pullen, Matthew C. Cane, Priyanka Prabhala, William Greenwald, Anke Schulte, Piero Marchetti, Mark Ibberson, Patrick E. MacDonald, Jocelyn E. Manning Fox, Anna L. Gloyn, Philippe Froguel, Michele Solimena, Mark I. McCarthy, and Guy A. Rutter

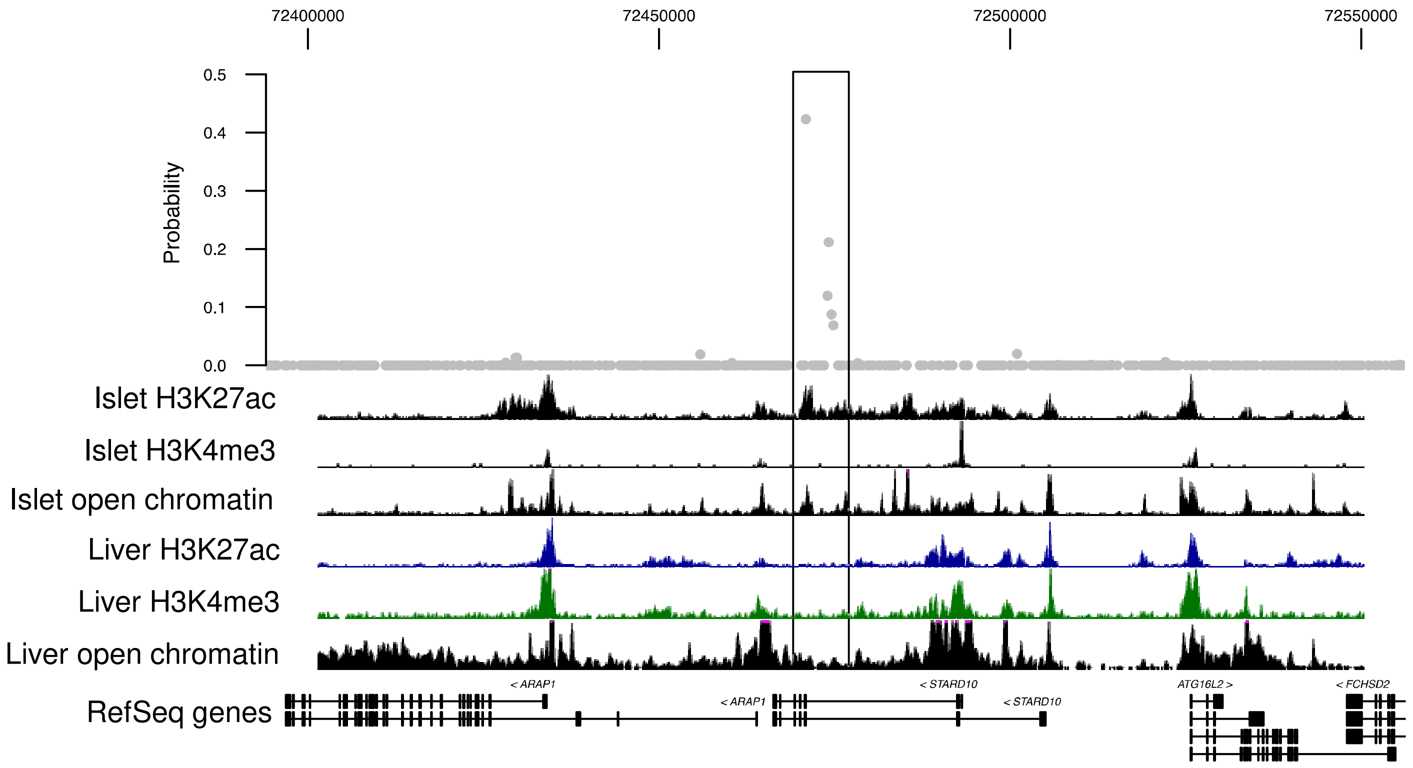
72400000
72450000
72500000
72550000
0.5
0.4
0.3
0.2
0.1
0.0
Probability
Islet H3K27ac
Islet H3K4me3
Islet open chromatin
Liver H3K27ac
Liver H3K4me3
Liver open chromatin
RefSeq genes
< ARAP1
< ARAP1
< STARD10
< STARD10
ATG16L2 >
< FCHSD2

Figure S1

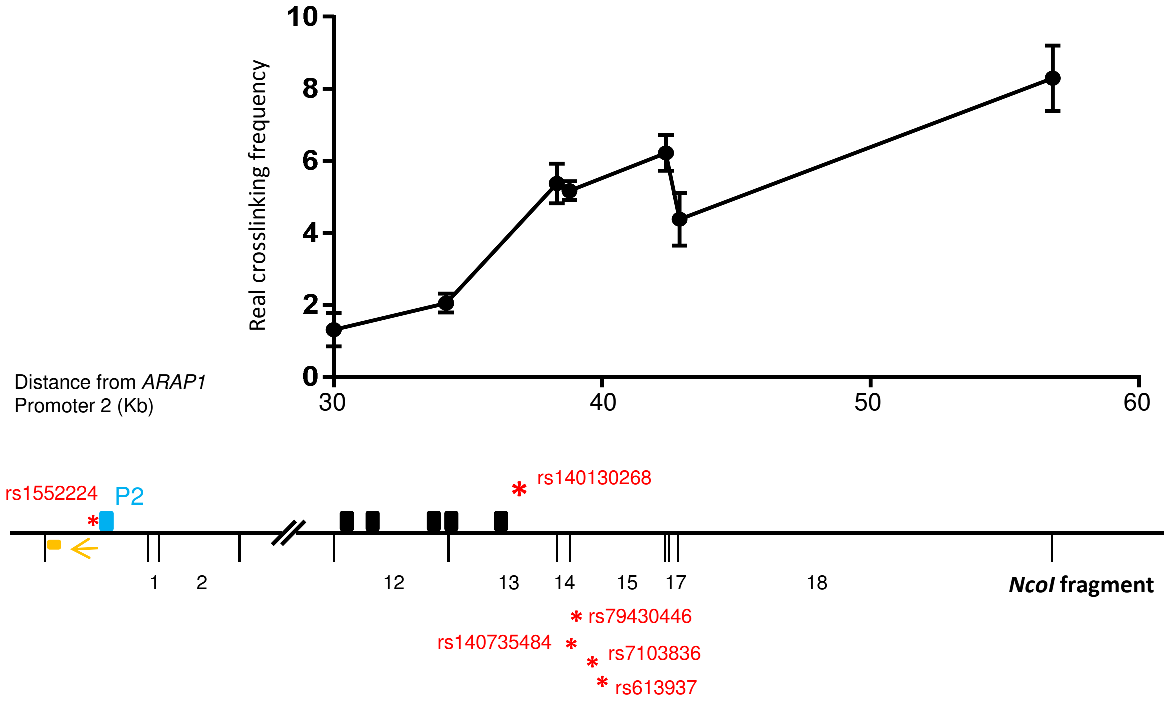
Real crosslinking frequency
10
8
6
4
2
0
30
40
50
60
Distance from ARAP1 Promoter 2 (Kb)
rs1552224
P2
rs140130268
1
2
12
13
14
15
17
18
NcoI fragment
rs79430446
rs140735484
rs7103836
rs613937

Figure S2

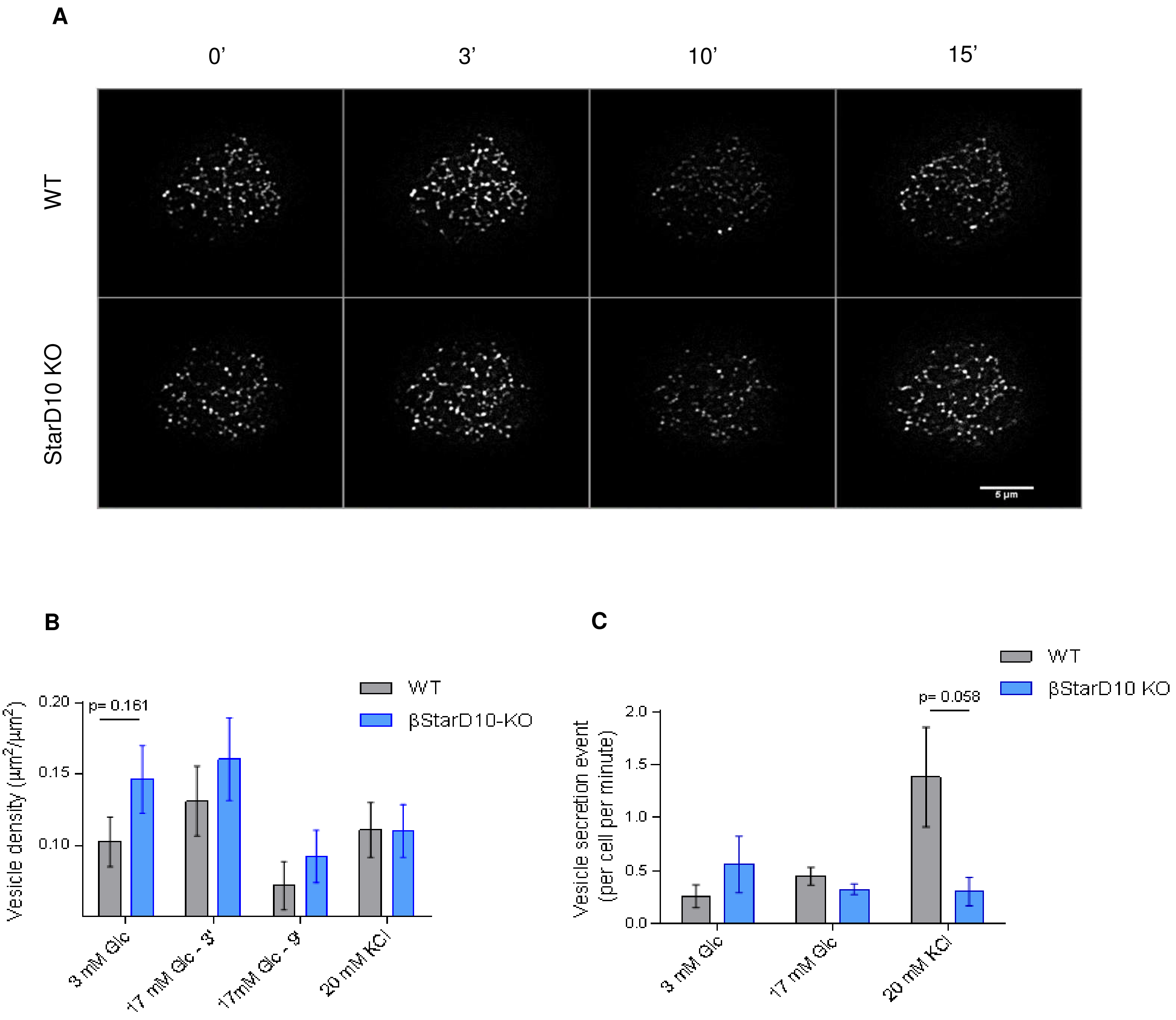
A
0’
3’
10’
15’
WT
StarD10 KO
5 µm
B
Vesicle density (µm²/µm²)
0.20
0.15
0.10
p= 0.161
WT
βStarD10-KO
3 mM Glc
17 mM Glc - 3'
17mM Glc - 9'
20 mM KCl
C
Vesicle secretion event (per cell per minute)
2.0
1.5
1.0
0.5
0.0
p= 0.058
WT
βStarD10 KO
3 mM Glc
17 mM Glc
20 mM KCl

Figure S3

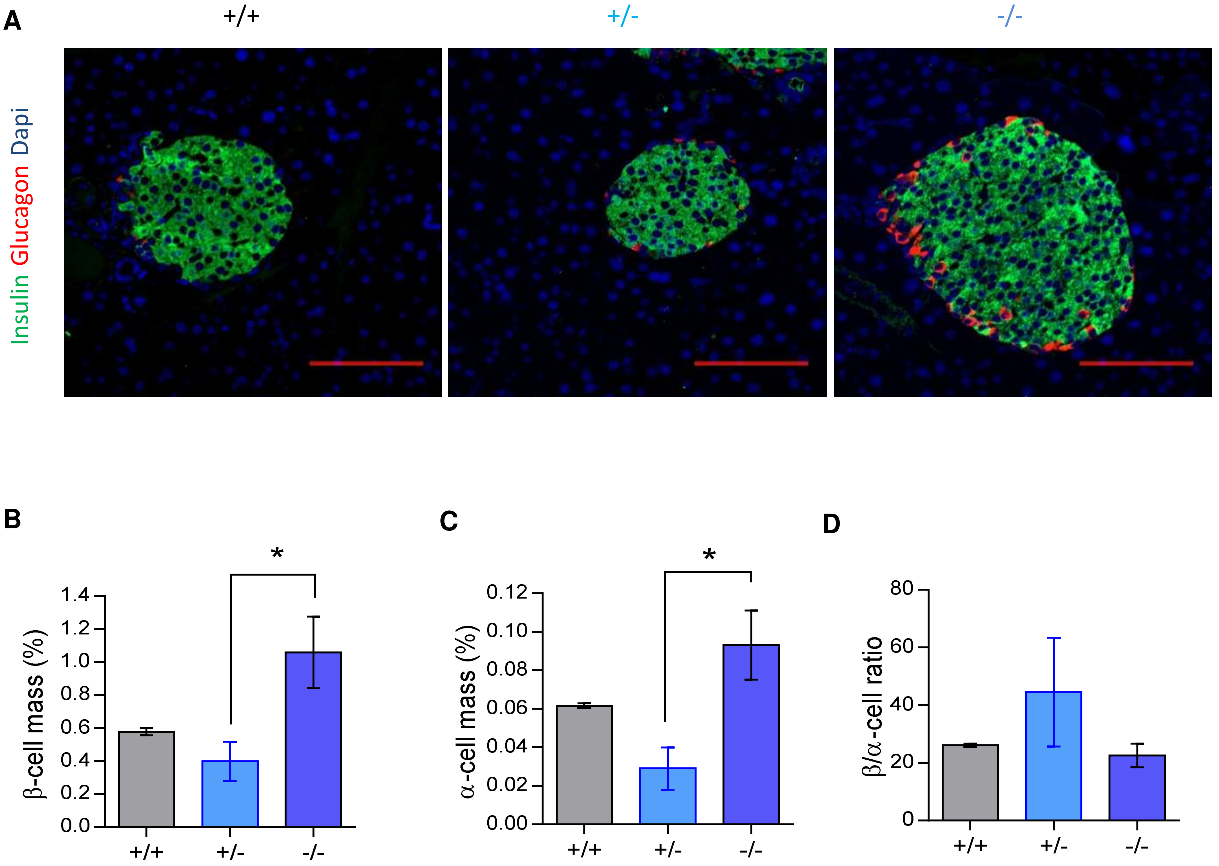
A
+/+
+/-
-/-
Insulin Glucagon Dapi
B
*
β-cell mass (%)
1.4
1.2
1.0
0.8
0.6
0.4
0.2
0.0
+/+
+/-
-/-
C
*
α-cell mass (%)
0.12
0.10
0.08
0.06
0.04
0.02
0.00
+/+
+/-
-/-
D
β/α-cell ratio
80
60
40
20
0
+/+
+/-
-/-

Figure S4

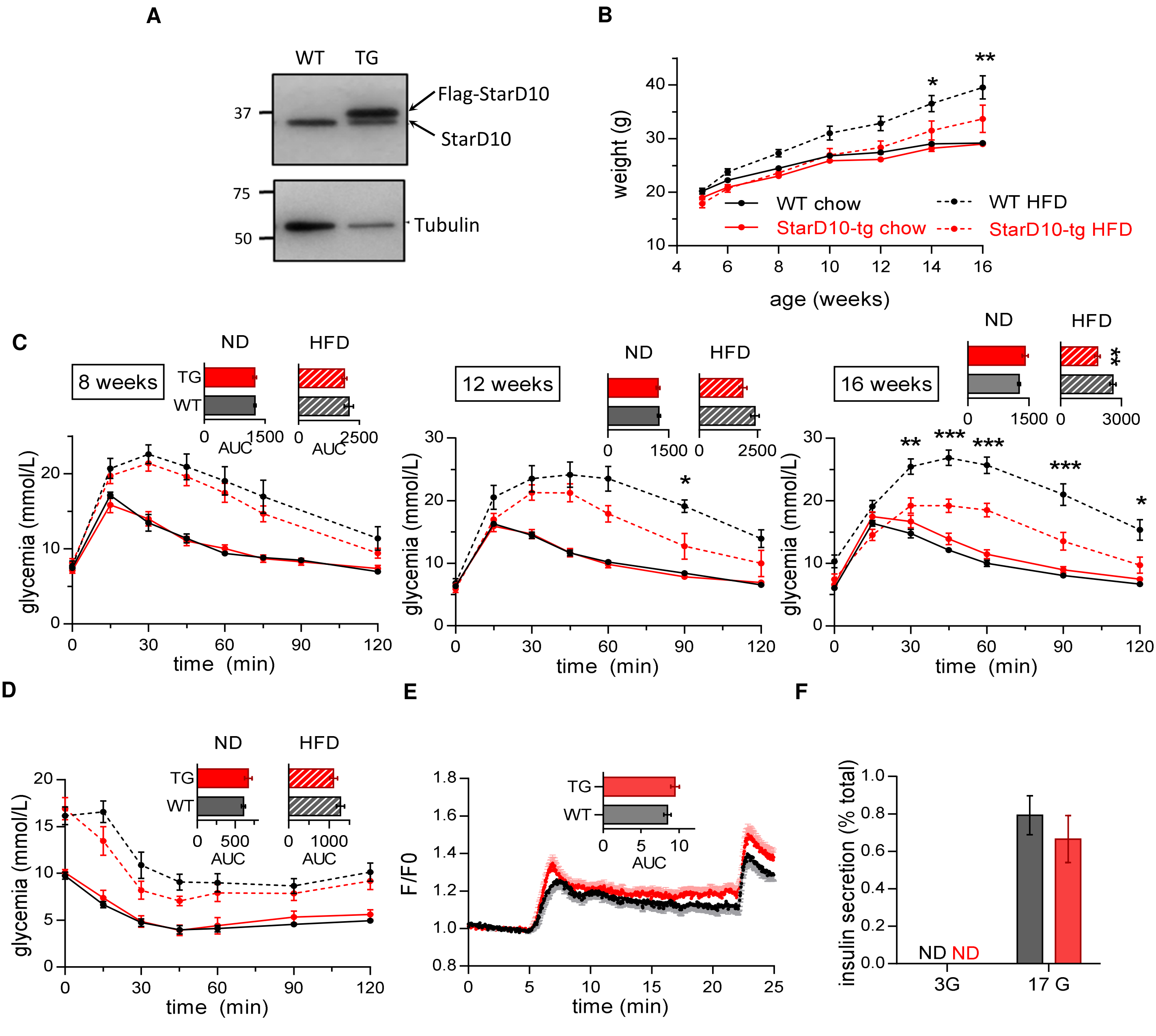

A
WT TG
37
Flag-StarD10
StarD10
75
50
Tubulin
B
weight (g)
age (weeks)
WT chow
StarD10-tg chow
WT HFD
StarD10-tg HFD
C
8 weeks
12 weeks
16 weeks
ND
HFD
TG
WT
AUC
glycemia (mmol/L)
time (min)
D
E
F/F0
time (min)
F
insulin secretion (% total)
ND ND
3G
17 G

Figure S5

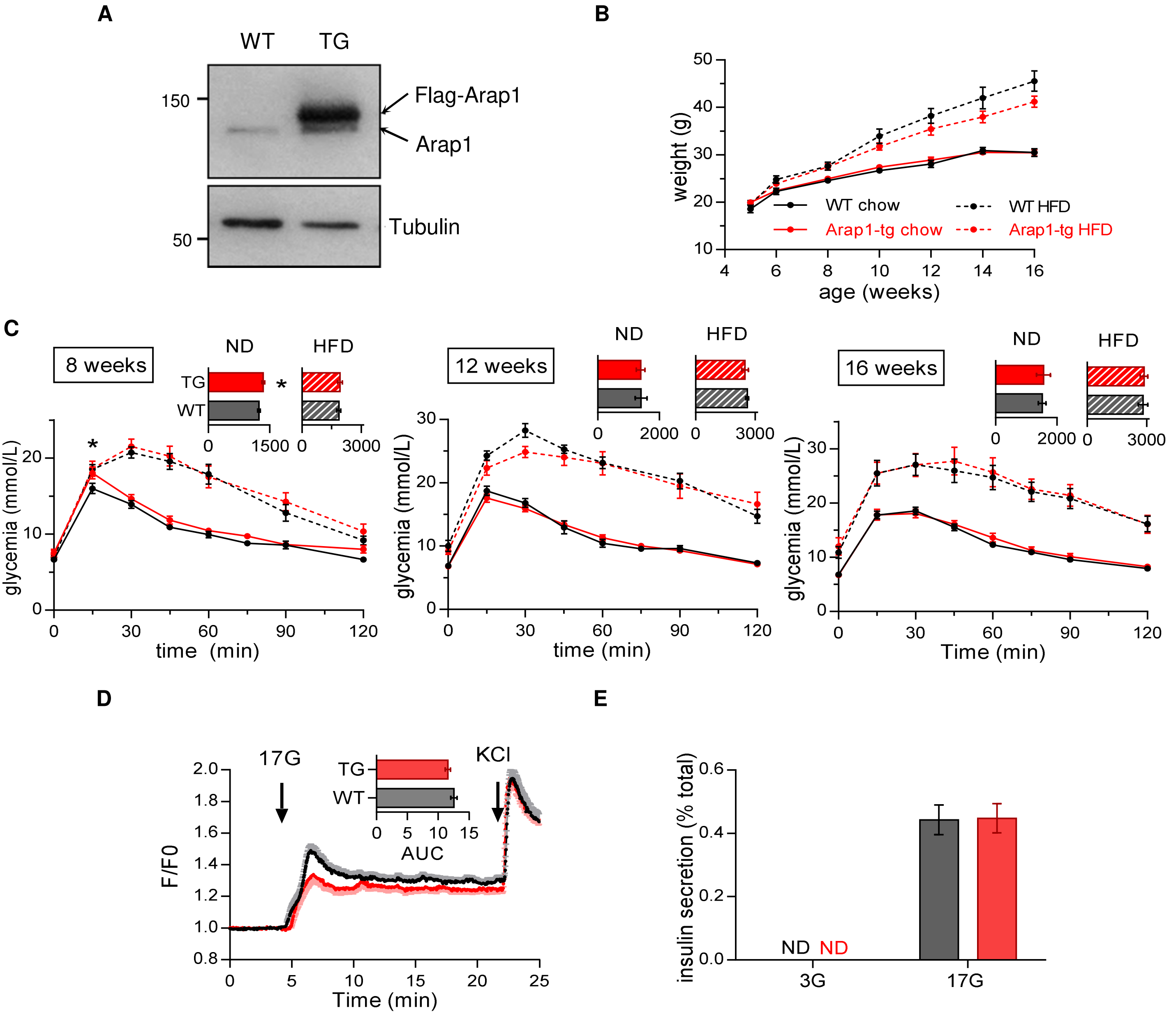
A
WT
TG
150
50
Flag-Arap1
Arap1
Tubulin
B
weight (g)
age (weeks)
WT chow
Arap1-tg chow
WT HFD
Arap1-tg HFD
C
8 weeks
12 weeks
16 weeks
ND
HFD
TG
WT
glycemia (mmol/L)
time (min)
Time (min)
D
17G
KCl
F/F0
AUC
Time (min)
E
insulin secretion (% total)
ND
ND
3G
17G

Figure S6

SUPPLEMENTAL DATA:

Supplemental figure legends:

Figure S1: **Epigenomic annotation at the *STARD10* locus**.

Top panel: posterior causal probabilities of each variant at the T2D signal. Bottom panel: raw signal from pancreatic islets and liver for H3K27ac, H3K4me3 and open chromatin (ATAC-seq or DNAase-seq). The variants most likely to be causal for the T2D signal (box) fall within a region of H3K27ac and open chromatin signal in islets, which are low for all assays in liver. Data are from ENCODE (The ENCODE Project Consortium, 2012)[1] and the NIH Roadmap 2015 (Roadmap Epigenomics Consortium, 2015) [2].

Figure S2: **A region carrying multiple risk variants is physically associated with the *ARAP1* promoter**.

3C-qPCR analysis of long-distance interactions at the *ARAP1-STARD10* locus assessed in human-derived EndoC-βH1 cells. The relative level of each ligation product (fragments 12 to 18) is plotted according to its distance (in Kb) from the *ARAP1* promoter 2 (P2). The constant primer and the Taqman probe are indicated in orange. Data were normalized to a *CXCL12* loading control. Blue box: *ARAP1* Promoter2, yellow box: qPCR probe; yellow arrow: qPCR constant primer; red stars: variants. The *NcoI* restriction fragments are indicated below the graph. *NcoI* fragments are numbered from fragment 1 to 18. The data represent the mean of three independent experiments.

Figure S3: **Secretory granule distribution and secretion assessed by TIRF Imaging**.

(A) Images were captured from single β-cells as described under "Supplemental methods" and show NPY-Venus-labelled granules before (0'), during (3') and after stimulation with 16.7 mM glucose. KCl (20 mM) was subsequently added at 12'. **(B)** Number of morphologically docked granules. Vesicle density was determined on top-hat filtered images as the surface occupied by vesicles on the total surface of the cell, and is related to the number of vesicles present at the membrane vicinity. Vesicle density was averaged on 120 images at 0, 3, 9 and 15 minutes for WT and β*StarD10* KO beta cells (*n*=6 and *n*=5 respectively). **(C)** Quantification of exocyotic events. Numbers of secretion events observable were counted per cell and per minutes for WT cells (n=5) and β*StarD10* KO cells (*n*=5) when imaged in 3mM Glc, 17mM Glc and 20mM KCl.

Figure S4: **Impact of *StarD10* deletion on β-cell mass**.

Pancreata from control (+/+), heterozygous (+/-) and homozygous (-/-) mice were fixed and subjected to immunohistochemical analysis for insulin and glucagon, as indicated. Representative islets are shown in (**A**; Scale bar: 100 µm). β-cell mass and α-cell mass represent the percentage of respectively insulin **(B)** and glucagon **(C)** positive staining area over the total pancreas section as measured using ImageJ software. β-cell/α-cell ratio was measured as the quantification of total insulin/glucagon positive staining on each pancreas section. **(D)** A total of five sections evenly separated by 25 µm in each pancreas was used (*p<0.05 vs +/-; *n*=3-4 animals in each group; One-way ANOVA, Tukey post-test).

Figure S5: **Improved glucose tolerance in *StarD10* transgenic mice.**

(A) Western blot analysis of *StarD10* expression showing the expression of the endogenous protein (lower band) and the flag-tagged transgene (upper band). **(B)** Growth curves of WT (black) and *StarD10* transgenic (red) male mice maintained on a regular chow diet (plain lines) or high fat diet (dotted lines) from 5 weeks of age. (*p<0.05, **p<0.01, WT vs StarD10-tg on high fat diet, two-way ANOVA, Sidak post-test). **(C)** Intraperitoneal glucose tolerance (1g/kg) was assessed at 8, 12 and 16 weeks of age in male mice. Areas under the curve are shown in inset. (*n*=5-11 animals per genotype. *p<0.05, **p<0.01, ***p<0.001, two-way ANOVA, Sidak post-test). **(D)** Intraperitoneal insulin tolerance was assessed at 17 weeks of age in mice fed a regular chow diet (0.75U/kg insulin), plain lines or a high fat diet (1U/kg), dotted lines. **(E)** Calcium responses to 17mM glucose and 20mM KCl of islets isolated from both male and female mice. **(F)** Insulin secretion assessed in islets isolated from both male and female, 19-week old WT (black) and littermate *StarD10* transgenic (red) mice on chow diet; ND: not detected; *n*=4-5 mice per genotype.

Figure S6: **No effect of *ARAP1* overexpression on glucose tolerance and insulin secretion in mice.**

(A) Western blot analysis of *Arap1* expression showing the expression of the endogenous protein (lower band) and the flag-tagged transgene (upper band). **(B)** Body weight of WT (black) and *ARAP1* transgenic (red) male mice maintained on a regular chow diet (plain lines) or high fat diet (dotted lines) from 5 weeks of age. **(C)** Intraperitoneal glucose tolerance (1g/kg) was assessed at 8, 12 and 16 weeks of age in male mice. Areas under the curve are shown in inset. *n*=9-12 animals per genotype. *p<0.05 (*: 15 min time point WT vs Arap1-tg, regular chow diet; two-way ANOVA, Sidak post-test). **(D)** Ca^{2+} responses of islets

isolated from both male and female mice to 17mM glucose and 20mM KCl. **(E)** Insulin secretion assessed in islets isolated from both male and female, 19-week old WT (black) and littermate *ARAP1* transgenic (red) mice on regular chow diet. ND: not detected. *n*=2 mice per genotype.

Table S1:

Primers for generating the *StarD10* expression construct

Primer name	Primer sequence
MmStarD10+Flag+NheI_F	tca**gctagc**atggactacaaggatgacgatgacaagGAAAAGCCAGCTGCCTCAACA
MmStarD10+XhoI_R	tca**ctcgag**TCAGGTGAGCGAGGTGTCAT

Primers for generating the *ARAP1* expression construct

HArap1+Flag+NheI_F	tca**gctagc**atggactacaaggatgacgatgacaagACCAAGAAGGAGGAGCCCCCA
HArap1+XhoI_R	tca**ctcgag**TCAGACGTTGCGCAGAAGAG

Genotyping primers:

StarD10 whole body KO mice:

STARD10-5arm-WTF	TGGGAAGCTCTTTGAGGGTA
Tm1a-5mut-R1	GAACTTCGGAATAGGAACTTCG
STARD10-Crit-WTR	TGGAAAATCAAATGCCCAAA

StarD10 conditional KO mice

STARD10-5arm-WTF	TGGGAAGCTCTTTGAGGGTA
STARD10-Crit-WTR	TGGAAAATCAAATGCCCAAA
Cre_F	CAGGCGTTTTCTGAGCATACC
Cre_R	CCGGTTATTCAACTTGCACCAT

StarD10 transgenic mice

StarD10_F	TGAGACTTTCGACATCGCCC
StarD10_R	CTTGGGACACCTCCAGGAAT
RIP7-rtTA_F	GGACGAGCTCCACTTAGACG
RIP7-rtTA_R	CAACATGTCCAGATCGAAATC

Arap1 conditional KO mice:

ARAP1-5arm-WTF	CCTCTTGGTCACTCCCATGT
ARAP1-Crit-WTR	ATCTCCACCCTCAACCTCCT
Cre_F	CAGGCGTTTTCTGAGCATACC
Cre_R	CCGGTTATTCAACTTGCACCAT

ARAP1 transgenic mice

ARAP1_F	TGCTTCTCAGACACGAACCA
ARAP1_R	CTTCCTCATCCACACTAAACACC
RIP7-rtTA_F	GGACGAGCTCCACTTAGACG
RIP7-rtTA_R	CAACATGTCCAGATCGAAATC

References

1. Gerstein MB, Kundaje A, Hariharan M, Landt SG, Yan KK, Cheng C, Mu XJ et al (2012) Architecture of the human regulatory network derived from ENCODE data. Nature 489 (7414):91-100

2. Kundaje A, Meuleman W, Ernst J, Bilenky M, Yen A, Heravi-Moussavi A, Kheradpour P et al (2015) Integrative analysis of 111 reference human epigenomes. Nature 518 (7539):317-330
